# Health care costs of rheumatoid arthritis: A longitudinal population study

**DOI:** 10.1371/journal.pone.0251334

**Published:** 2021-05-06

**Authors:** Mark Tatangelo, George Tomlinson, J Michael Paterson, Edward Keystone, Nick Bansback, Claire Bombardier

**Affiliations:** 1 University of Toronto, Toronto, Ontario, Canada; 2 University Health Network, Toronto, Ontario, Canada; 3 ICES, Toronto, Ontario, Canada; 4 University of British Columbia, Vancouver, British Columbia, Canada; Qatar University, QATAR

## Abstract

Quantifying the contribution of rheumatoid arthritis to the acquisition of subsequent health care costs is an emerging focus of the rheumatologic community and payers of health care. Our objective was to determine the healthcare costs before and after diagnosis of rheumatoid arthritis (RA) from the public payer’s perspective. The study design was a longitudinal observational administrative data-based cohort with RA cases from Ontario Canada (n = 104,933) and two control groups, matched 1:1 on year of cohort entry from 2001 to 2016. The first control group was matched on age, sex and calendar year of cohort entry (diagnosis year for those with RA); the second group added medical history to the match before RA diagnosis year. The main exposure was new onset RA. The secondary exposure was calendar year of RA diagnosis to compare attributable costs over the study observation window. Main outcomes were health care costs in 2015 Canadian dollars, overall and by cost category. We used attribution methods to classify costs into those associated with RA, those associated with comorbidities, and age/sex-related underlying costs. Health care costs associated with RA increased up to the year of diagnosis, where they reached $8,591: $4,142 in RA associated costs; $1,242 in RA comorbidity associated costs; and $3,207 in underlying costs. In the eighth-year post diagnosis, the RA costs declined to $2,567 while the RA comorbidity associated costs remained relatively constant at $1,142, and the underlying age/sex related cost increased to $4,426. RA patients had lower costs when diagnosed in later calendar years. Our results suggest a large proportion of disease related health care costs are a result of costs associated with RA comorbidities, which may appear many years before diagnosis.

## Introduction

The association of rheumatoid arthritis (RA) with long-term health care costs are unclear and difficult to measure because of the unique treatment pathway of this complex chronic disease requiring long-term medication therapy [1,2]. Clinical trials of medications used in RA are highly selective with only 8% of patients seen in usual care meeting eligibility criteria [3–5]. Because of their design and short follow-up, clinical trials cannot capture the long-term costs of RA that accrue over many years or the additional costs of RA patients that may arise years before diagnosis. Therefore, research on the health care costs of RA have usually relied on modeled projections of future costs from clinical trials, or data from observational studies [6–9].

Observational studies of the costs of RA have had design limitations, including small or non-representative samples, data from multiple-insurers, limited follow-up time, patient-reported costs, and failure to adjust for secular trends in medical costs over time. A commonly used method in disease costing studies is to match diseased individuals with non-diseased controls on age/sex and sometimes geographic location [10–15] with the difference in costs between diseased and matched controls attributed to the condition or disease. This method could potentially misattribute some costs to RA because it does not capture pre-existing differences in medical history between RA patients and non-diseased controls. Other limitations of existing costing studies are their inability to separate health care costs into those associated with RA, costs associated with comorbidities arising from RA, and underlying costs that would have occurred irrespective of an RA diagnosis [6,7,16,17].

Our primary objective was to measure the health care costs associated with RA before and after diagnosis from the payer’s perspective among a population with identical public health insurance coverage. A secondary objective was to measure if the health care costs associated with RA changed over time since the introduction of biologic therapies.

## Materials and methods

### Statistical attribution mechanisms for the health care costs of RA

**Fig 1:**
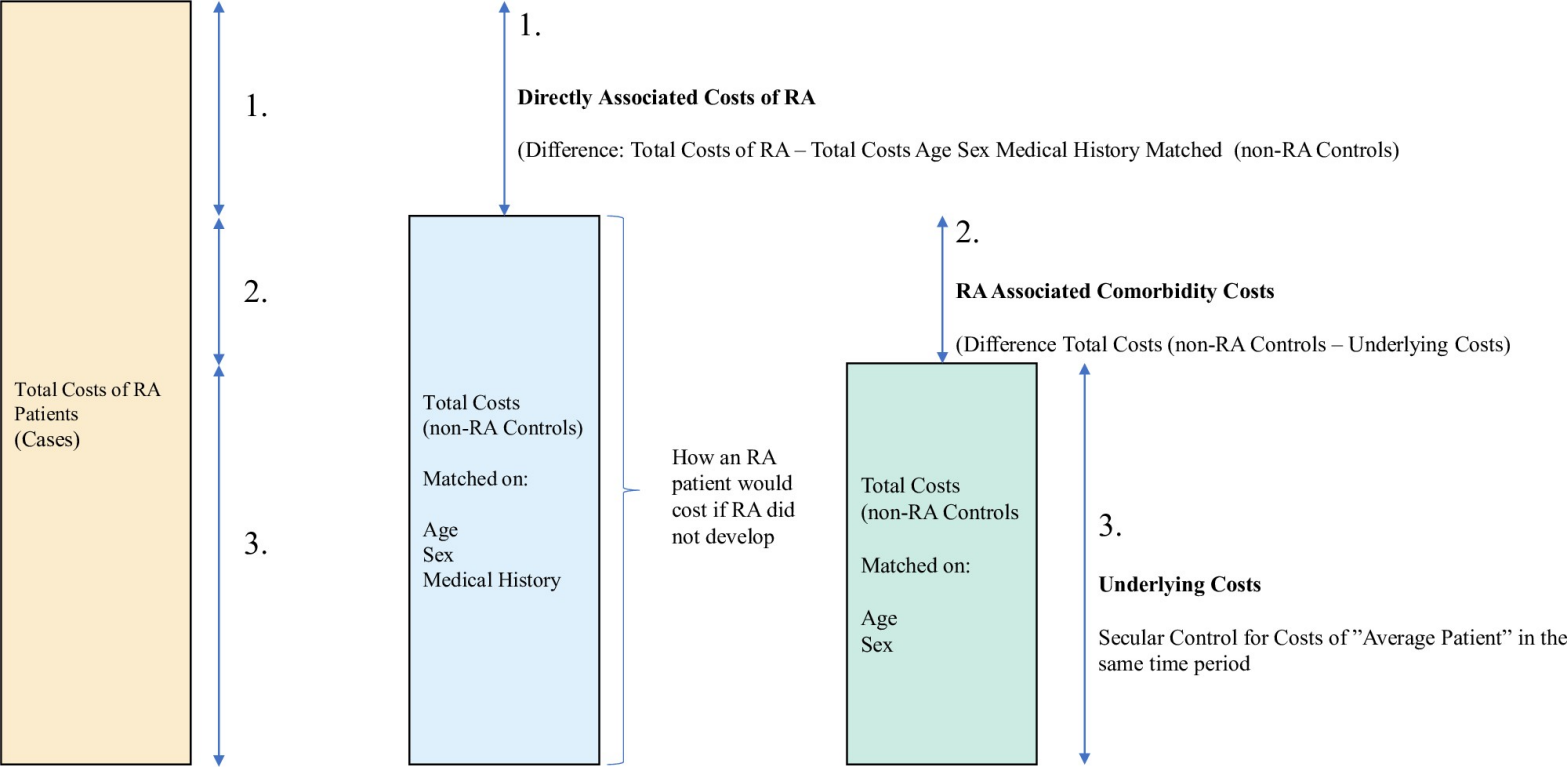
Statistical attribution mechanisms for the health care costs of RA. No legend.

Health care costs of RA can be divided into 3 parts (Fig 1): 1) The directly associated costs of RA which can be calculated by subtracting the costs of non-RA controls from the costs of RA cases. 2) The RA associated comorbidity costs which can be calculated by subtracting the underlying costs from the costs of non-ra controls matched on medical histories. 3) Underlying costs which are a secular control for the costs of an “Average Patient” in the same time period.

The RA related costs are the marginal costs of RA alone, which can be calculated by separating out the costs of conditions that are more likely to occur as a result of RA. These costs are ascertained by measuring the costs of an RA case and subtracting the costs of a patient with a similar medical history, but no RA diagnosis. The costs of RA comorbidities are costs that occur as a result of subsequent conditions arising from RA. For example, an RA comorbidity associated cost could be increased cardiovascular events as a result of RA related inflammation.

This study was approved by the University Health Network Research Ethics Board. All analyses were conducted in R 3.4.1 [18], SAS Enterprise 7.0 [19] on Redhat Linux 7 [20] with IBM LSF [21] and SLURM workload management software [22].

### Population

RA cases were residents of Ontario, Canada for at least one year who were eligible for identical, single-payer provincial health insurance coverage, and aged 15 years or older at RA diagnosis at any point from 2001 to 2016 inclusive (n = 104,933). The year of RA diagnosis was determined using a validated algorithm (2 RA related physician visits < = 2 years apart with at least 1 visit to a musculoskeletal specialist or 1 hospitalization for RA) [23]. Eligible controls received insured single-payer health care but did not have RA. Data were housed and analyzed at ICES (formerly the Institute for Clinical Evaluative Sciences) [24], an independent health services research institute funded by the Ontario Ministry of Health (www.ices.on.ca) which contains administrative billing records for medical services in Ontario.

### Year of RA diagnosis (primary exposure)

The primary exposure was the year of RA diagnosis, defined as the year patients met the validated administrative data case definition for RA in each year from 2001–2016.

### Changes in costs by calendar year (secondary exposure)

To evaluate changes in health care costs over time, attributable costs were also calculated for each calendar year of RA diagnosis. The introduction of new treatments and improvements in care forms a natural experiment because exposure to new treatments and care patterns increases over time. Patients diagnosed in later calendar years would be exposed to newer treatments sooner in their disease course than patients diagnosed in earlier calendar years and patients who lived until the new treatments were available would receive partial exposures to new treatment options. We hypothesized that use of diagnosis year as a measure of exposure to new treatments would reveal an association of diagnosis year with changes in health care costs.

### Cost outcomes

Cost outcomes were total health care cost per patient per year in 2015 Canadian dollars (CAD) from the public payer’s perspective [25]. In Ontario most medical services are paid for by a single publicly funded insurer, the Ontario Ministry of Health. Funded services include all physician visits, hospital care, emergency department visits, and prescription medications among patients over the age of 64 or those receiving social assistance. Costs not included are drug costs for those under age 65 except those on social assistance, or with medication expenses exceeding 4% of net income. Other uninsured health care costs are chiropractors, elective surgeries, and non-evidence informed treatments or procedures. Costs were aggregated and reported yearly at 5, 2, and 1 year before the year of RA diagnosis, the year of RA diagnosis, and 1, 2, 5, and 8 years after RA diagnosis.

Results were reported as total annual costs and split by 17 costing categories (S1 File). Individual events were costed for fee-for service physician billings, non-physician billings, and laboratory billings from the Ontario Health Insurance Plan (OHIP) database. Physician services in capitation models were costed by applying payments weighted by age and complexity criteria. Medication costs were measured at the prescription level (list prices) from the Ontario Drug Benefits Program for all prescriptions after age 65, and for prescriptions exceeding 4% of a patient’s after-tax income below age 65 or recipients of social assistance. Inpatient hospitalizations, emergency department visits, and same day surgery were costed using resource intensity weighting, with oncology, dialysis, and hospital outpatient clinic visits derived using an analogous ambulatory care weighting system [26]. Complex continuing care, inpatient mental health, and rehabilitation costs [27,28] were calculated based on length of stay and case mix [29], while long-term care and home care services were costed using average unit costs of service per hour or day [30]. All unit costs and weighting values were obtained from the Ontario Ministry of Health and Long-Term Care and the Canadian Institute for Health Information, these datasets were linked using unique encoded identifiers and analyzed at ICES.

#### Analysis of total health care cost outcomes before RA diagnosis

Emerging evidence suggest that the negative inflammatory effects of RA can often manifest before the confirmation of an RA diagnosis. Therefore, we also measured the differences in health care costs before RA diagnosis between RA patients and their matched controls.

#### Matching covariates

Clinical covariates from administrative data were included and were measured annually (S 1). These covariates were 27 Major Expanded Diagnosis Clusters (MEDC), which were derived from the Johns Hopkins Adjusted Clinical Group System^®^ Version 10 [31]. The MEDC methodology assigns diagnostic codes found in physician claims, the hospital Discharge Abstract Database, and the National Ambulatory Care Reporting System (NACRS) [32]. The NACRS dataset contains data for all hospital-based day surgery, outpatient cancer and dialysis services, and emergency department services. The Ontario Marginalization Index and neighbourhood income quintile were measured and reported but controls were not matched on these covariates [33].

### Statistical analysis

#### Study design

Our study was a matched longitudinal cohort using individual-level administrative data

#### RA cases

RA cases Table 1 were matched to two control groups each with a 1:1 case-to-control ratio to provide each case with an eligible control in each control group.

**Table 1:** Patient characteristics and costs at year of diagnosis for RA cases, index year for non-RA controls.

Characteristic	RA Cases (n = 104,933)	Age/Sex/Medical History Matched Controls (n = 103,853)	Average Difference (Δ)	Age/Sex Matched Controls (n = 104,333)	Average Difference (Δ)	RA Associated Costs	RA Associated Comorbidity Costs	Underlying Costs
Age (Mean, Median, SD, Range)	56.8,58,16.7, (15–99)	56.8,58,16.7, (15–99)	0	56.8,58,16.7, (15–99)	0	-	-	-
Female Sex %, n	69.6, 73047	69.6, 73047	0	69.6, 73047	0	-	-	-
Year of Diagnosis (Mean, Median, SD, Range)	2006,2007,6.3, 2001–2016	-	0	-	0	-	-	-
Income Quintile (Mean, Median, Q1, Q3, Range)	3.005,3,2,4	2.99, 3, 2, 4	0.01	3.00, 3, 2, 4	<0.01	-	-	-
Rurality Score (Mean, Median, Q1, Q3)	11.25,2,0,17	10.29, 2, 0, 12	0.96	10.06, 2, 0, 11	1.19	-	-	-
Ontario Marginalization Score Summary (Mean, Median, Q1, Q3)	3.037, 3, 2.5, 3.75	3.086, 3, 2.5, 3.75	<0.01	3.087, 3, 2.5, 3.75	-0.05	-	-	-
Material Deprivation (Mean, Median, Q1, Q3)	3.012, 3, 2, 4	3.036, 3, 2, 4	-0.02	3.02, 3, 2, 4	<0.01	-	-	-
Dependency (Mean, Median, Q1, Q3)	3.064, 3, 2, 4	3.038, 3, 2, 4	<0.01	3.008, 3, 2, 4	0.06	-	-	-
Housing Instability (Mean, Median, Q1, Q3)	3.057, 3, 2, 4	3.094, 3, 2, 4	-0.04	3.115, 3, 2, 4	-0.06	-	-	-
Ethnic Concentration (Mean, Median, Q1, Q3)	3.016, 3, 2, 4	3.176, 3, 2, 4	-0.16	3.207, 3, 2, 5	-0.19	-	-	-
Total Healthcare Costs^b^	8,591	4,449	4,142	3,207	5,384	4,142	1,242	3,207
Inpatient Care	2,672	926	1,746	651	2,021	1,746	275	651
Physician Fee for Service Billings (Specialist)	1,656	704	952	509	1,147	952	195	509
Drug Benefits	958	641	317	475	482	317	166	475
Outpatient	618	249	368	183	434	369	66	183
Physician Fee for Service Billings (General Practitioner)	414	273	141	194	219	141	79	194
Laboratory	307	118	189	89	218	189	29	89
Home Care Services	410	237	173	168	242	173	69	168
Rehabilitation	297	81	216	59	238	216	22	59
Emergency Department	271	135	136	90	181	136	45	90
Same Day Surgery	202	141	61	107	96	61	34	107
Complex and Continuing Care	171	143	28	74	97	28	69	74
Long-Term Care	157	370	-213	295	-138	-213	75	295
Capitation Costs (Family Health Teams)	100	87	13	79	21	13	8	79
Cancer Clinics	83	80	3	75	7	3	5	75
Dialysis Clinics	83	92	-9	47	36	-9	45	47
Mental Health Inpatient Care	50	86	-37	44	6	-36	42	44
Assistive Devices	15	10	5	7	8	5	3	7

^b^All costs are presented as 2015 Inflation Adjusted Canadian Dollars per-patient per year.

#### Age sex matched controls

Potential age and sex matched controls were all those members of the population who were free of the algorithm-defined RA diagnosis before and during the year of diagnosis of the index case Table 1. Controls were sampled with replacement from the eligible pool and the index date assigned to controls was the year of RA diagnosis (2001–2016) of their corresponding matched case.

#### Age sex medical history matched controls

**Fig 2:**
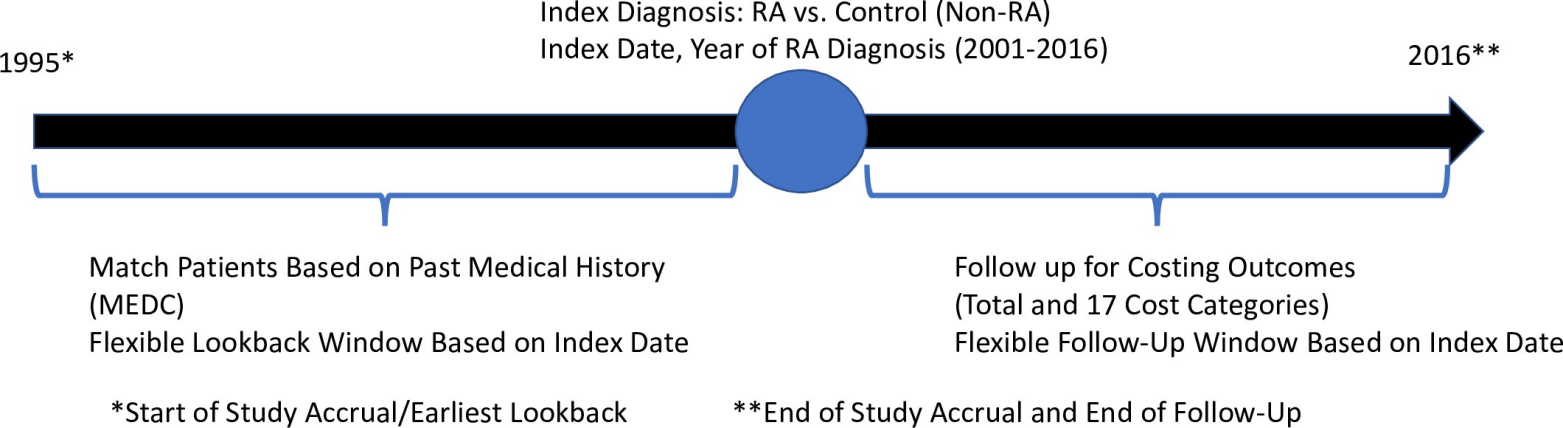
Study diagram of flexible study accrual dates, medical history lookback and outcome follow-up. No legend.

The second set of controls was matched to RA cases on age, sex with an identical methodology described above. In addition to age and sex matching, the second control group added to the match all available years of medical history before the year of RA diagnosis Table 1. Each of the 16 year-of-diagnosis cohorts (2001–2016), had a different length of lookback duration for establishing medical history and a different subsequent duration of cost accrual (Fig 2). Medical history was operationalized with balanced risk set matching using Mahalanobis distance to create a control group matched on similar medical history based on clinical covariates [34–40]. This technique uses clinical covariates (MEDC), normalizes them on a common scale and give each case a pairwise "similarity score" to each potential control. The more similar a case is to a control, the closer the similarity score will be to 0, with controls selected on the minimum Mahalanobis distance for each RA case within the age/sex/year stratum [35,37,38,41]. This level of matching ensures similarity between the groups on clinical conditions other than RA, controls for secular changes in health care costs over time and reduces the potential for conditions other than RA to account for differences in health care costs. (Fig 2).

### Analysis of costs of RA before and after diagnosis

**Fig 3:**
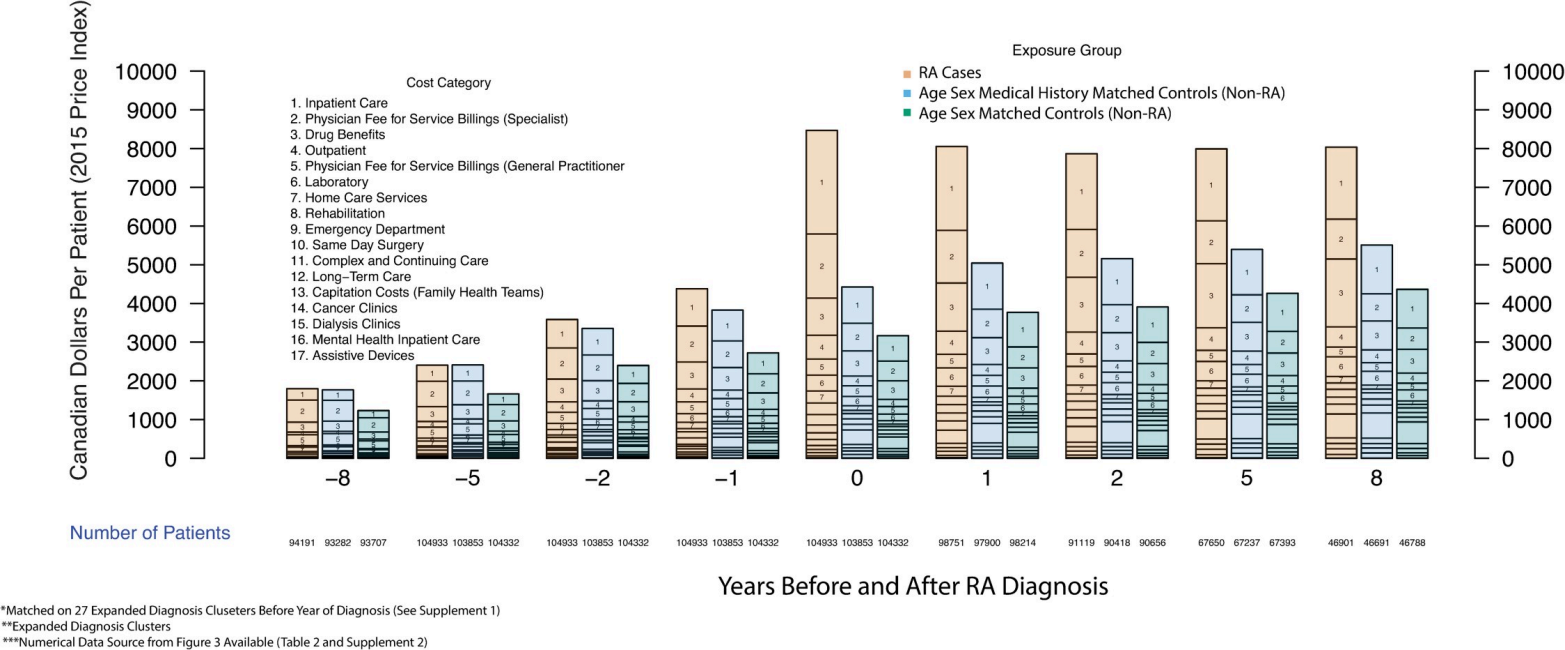
Annual per-patient health care costs over time grouped by year before and after rheumatoid arthritis diagnosis (public payer’s perspective). X-axis: Years before and after RA diagnosis, Y-axis: Canadian Dollars Per Patient (2015 Price Index).

Differences were calculated between RA cases and each of the two matched controls for total health care costs (Table 2) and for 17 cost categories (Fig 3, S1 Table, S2 File).

**Table 2:** Total patient outcomes at year of diagnosis and 1, 2, 5, and 8-Year pre-and post-diagnosis (Data source for Fig 3).

Years Before / After Diagnosis	RA Cases (2015 CAD/patient)	Age/Sex/Medical History Matched Controls (2015 CAD/patient)	Average Difference	Age/Sex Matched Controls (2015 CAD/patient)	Average Difference (2015 CAD/patient)	Direct RA Associated Costs (2015 CAD/patient)	Indirect RA Associated (2015 CAD/patient)	Age/Sex Related Costs (2015 CAD/patient)
(n pairs of cases and controls)			(2015 CAD/patient)					
-8 (n = 94,216)	1853	1797	56	1262	591	56	535	1262
-5 (n = 104,933)	2462	2435	27	1689	773	27	746	1689
-2 (n = 104,933)	3656	3379	277	2430	1226	277	949	2430
-1 (n = 104,933)	4460	3853	607	2758	1702	607	1095	2758
0 (n = 104,933)	8591	4449	4142	3207	5384	4142	1242	3207
1 (n = 98,780)	8163	5075	3088	3812	4351	3088	1263	3812
2 (n = 91,146)	7971	5195	2776	3952	4019	2776	1243	3952
5 (n = 67,672)	8087	5441	2646	4310	3777	2646	1131	4310
8 (n = 46,916)	8135	5568	2567	4426	3709	2567	1142	4426

### Analysis of costs of RA before and after diagnosis by calendar year of diagnosis

**Fig 4:**
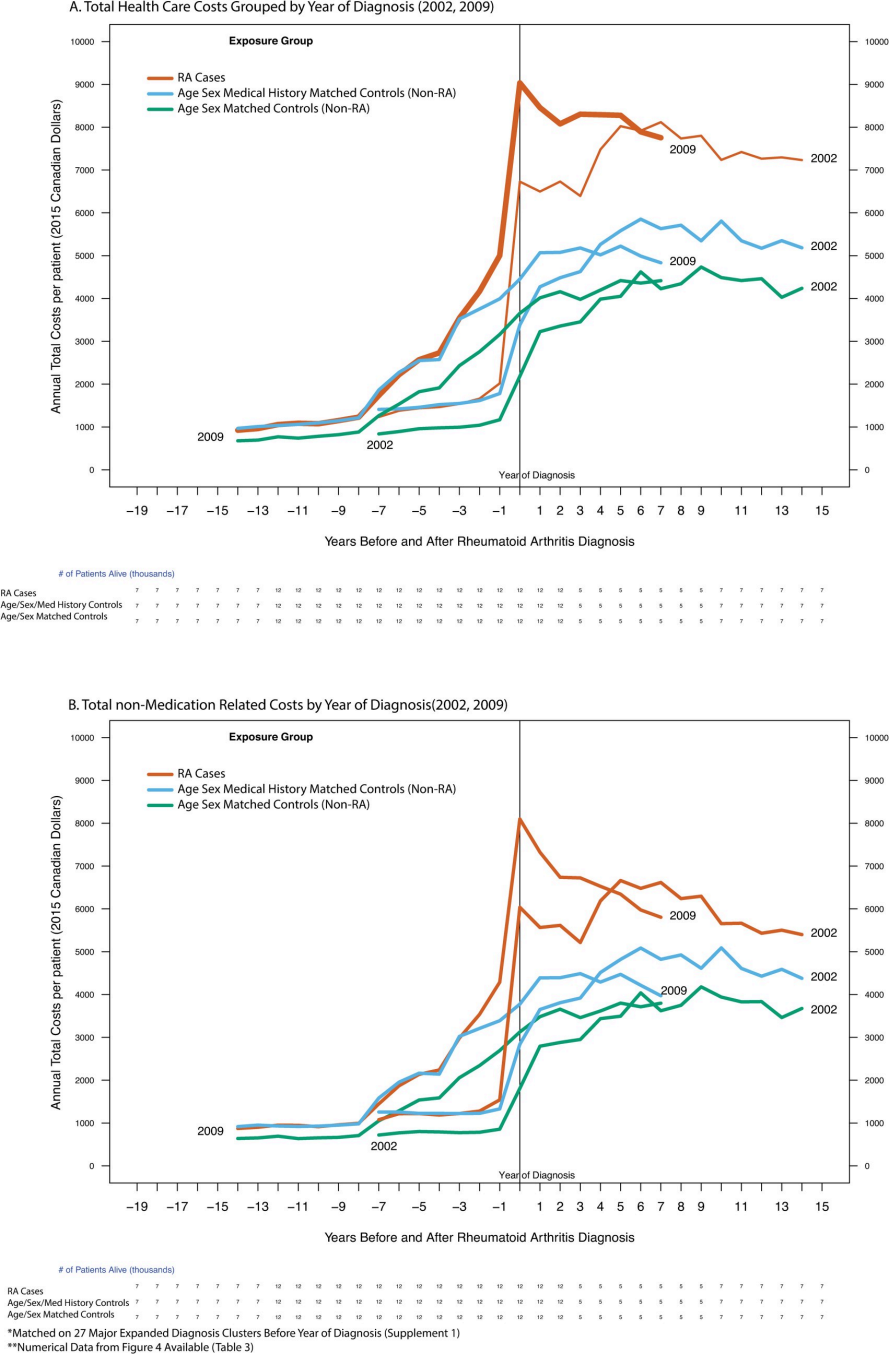
Annual per-patient health care costs over time grouped by diagnosis year (2002, 2009) before and after rheumatoid arthritis diagnosis (public payer’s perspective). X-axis: Years before and after RA diagnosis, Y-axis: Annual Costs Per Patient (2015 Canadian Dollars), Grouping Variable: Exposure Group).

**Fig 5:**
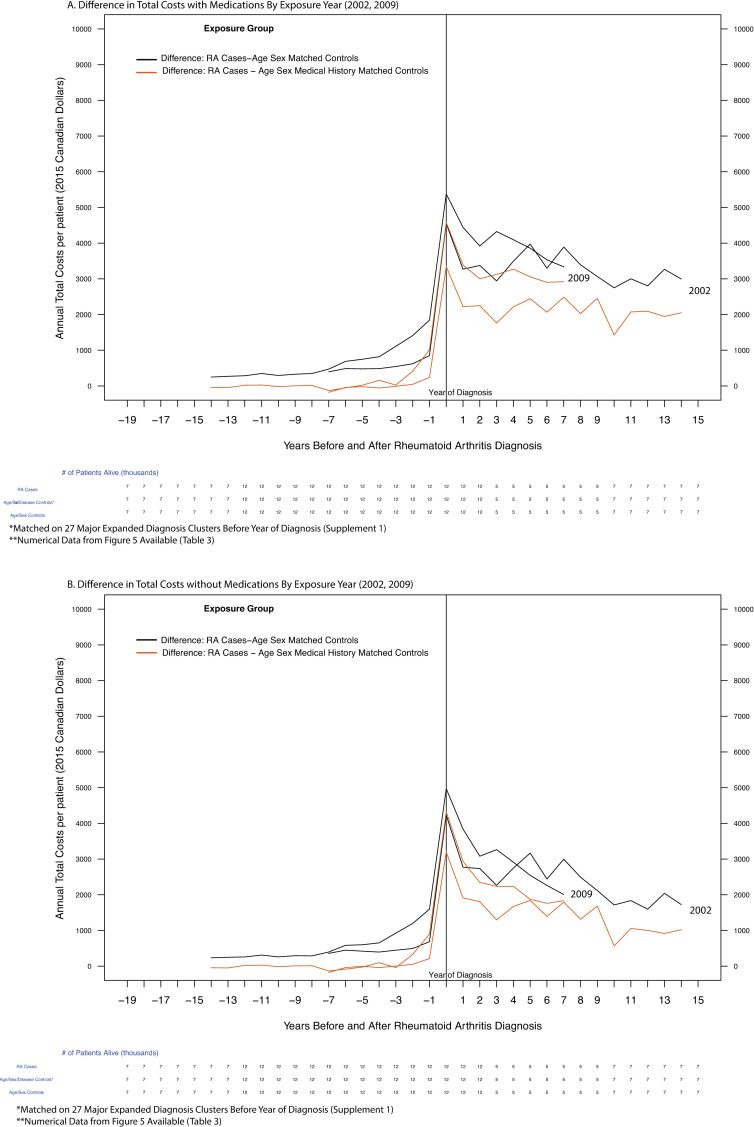
Difference in Annual per-patient health care costs over time grouped by diagnosis year (2002, 2009) before and after rheumatoid arthritis diagnosis (public payer’s perspective). X-axis: Years before and after RA diagnosis, Y-axis: Annual Costs Per Patient (2015 Canadian Dollars), Grouping Variable: Exposure Group).

Costs were also calculated by year-of-RA-diagnosis cohort (and presented for 2002 and 2009), at 1, 2, 5 before and after RA diagnosis (Figs 4 and 5., Tables 3 and S2). Changes in costs over time by calendar year of diagnosis were examined with and without the inclusion of medication costs. The cost differences between cases and controls for the 2002 cohort were compared to the differences for the 2009 cohort at 1, 2, 5, years post diagnosis (Table 3).

**Table 3:** Patient outcomes by year of exposure with and without medication costs (Data source for Fig 4).

Incident Year	Years Before / After RA Diagnosis (n pairs), n biologics	RA Cases(2015 CAD/patient)	Age/Sex/Medical History Matched Controls (2015 CAD/patient)	Average Difference (2015 CAD/patient)	Sum of the Difference in Costs Since Diagnosis	Age/Sex Matched Controls (2015 CAD/patient)	Average Difference (2015 CAD/patient)	Sum of the Difference in Costs Since Diagnosis	Direct RA Associated Costs	Indirect RA Associated	Age/Sex Related Costs	RA Cases (2015 CAD/patient)	Age/Sex/Medical History Matched Controls (2015 CAD/patient)	Average Difference (2015 CAD/patient)	Sum of Difference in Costs Since Diagnosis	Age/Sex Matched Controls (2015 CAD/patient)	Average Difference (2015 CAD/patient)	Sum of Difference in Costs Since Diagnosis	Direct RA Associated Costs	Indirect RA Associated	Age/Sex Related Costs
	A. Total Costs with Medications											B. Total Costs without Medications									
2002	-8 (n = 5,322),	-	-	-	-	-	-	-	-	-	-	-	-	-	-	-	-	-	-	-	-
	0																				
	-5 (n = 5,322),	1503	1522	-19	-3422	1007	496	-4129	-19	515	1007	1287	1289	-2	-3258.5	849	438	-3874	-2	440	849
	0																				
	-2 (n = 5,322),	1735	1685	50	-3353	1086	649	-3967	50	599	1086	1353	1294	59	-3198	829	523	-3789	59	465	829
	0																				
	-1 (n = 5,322),	2109	1855	254	-3151	1218	891	-3736	254	637	1218	1632	1405	227	-3030	906	725	-3587	227	499	906
	0																				
	0	6870	3466	3404	0	2243	4627	0	3404	1223	2243	6178	2921	3257	0	1867	4312	0	3257	1054	1867
	(n = 5,322), <8																				
	1	6617	4360	2257	-1147	3297	3320	-1306	2257	1063	3297	5686	3739	1947	-1310	2865	2820	-1492	1947	874	2865
	(n = 5,322), 11																				
	2	6851	4576	2275	-1129	3436	3415	-1211	2275	1140	3436	5736	3901	1835	-1422	2960	2275	-2037	1835	941	2960
	(n = 5,322), 24																				
	5	8095	5630	2465	-939	4106	3989	-636	2465	1524	4106	6735	4870	1865	-1392	3549	3186	-1126	1865	1321	3549
	(n = 5,322), 31																				
	8	7829	5792	2037	-1366	4428	3401	-1225	2037	1364	4428	6334	5006	1328	-1929	3833	2501	-1811	1328	1173	3833
	(n = 5,322), 27																				
2009	-8	1294	1271	23	-4609	926	368	-5060	23	345	926	1059	1038	21	-4350.4	751	309	-4713	21	287	751
	(n = 6637)																				
	-																				
	-5 (n = 6637), <8	2653	2618	35	-4597	1877	776	-4653	35	741	1877	2219	2230	-11	-4384	1591	628	-4394	-11	639	1591
	-2 (n = 6637), <8	4215	3790	425	-4207	2793	1422	-4007	425	997	2793	3586	3245	341	-4030	2377	1209	-3813	341	868	2377
	-1 (n = 6637),	5066	4032	1034	-3598	3202	1864	-3565	1034	830	3202	4352	3430	922	-3450	2734	1618	-3404	922	696	2734
	8																				
	0	9136	4504	4632	0	3707	5429	0	4632	797	3707	8201	3828	4373	0	3179	5022	0	4373	649	3179
	(n = 6637), 20																				
	1	8563	5136	3426	-1206	4080	4483	-946	3427	1056	4080	7430	4458	2972	-1400	3548	3882	-1140	2972	910	3548
	(n = 6637), 45																				
	2	8198	5158	3040	-1592	4235	3963	-1466	3040	923	4235	6859	4475	2384	-1988	3735	3124	-1898	2384	740	3735
	(n = 6637), 91																				
	5	8373	5293	3080	-1553	4482	3890	-1539	3080	811	4482	6440	4544	1896	-2476	3864	2577	-2445	1896	680	3864
	(n = 6637), 66																				
	8	-	-	-	-	-	-	-	-	-	-	-	-	-	-	-	-	-	-	-	-

## Results and discussion

### Patient population

From 2001 to 2016, 104,933 patients were diagnosed with RA (Table 1). All RA cases were matched, giving two non-RA control groups. The total pool of available controls for matching was 18.6 million residents eligible for health care within the study window.

At the year of diagnosis, RA cases were predominantly female (69.6%, n = 73,047), with a mean age of 56.8 years, (median = 58, SD = 16.7, Range = 15–99), mean year of diagnosis 2009, (median = 2009). RA patients and matched controls had similar demographic characteristics rurality score (median = 2), and Ontario Marginalization Index composite score (Table 1).

### Aggregate costs before and after RA diagnosis

RA cases cost more in health care service use than patients matched on age/sex alone with cost differences increasing from $590 per patient 8-years before diagnosis to a peak difference of $5,384 per patient in the year of diagnosis and declining to $3,708 per patient 8-years after diagnosis (Fig 3, Tables 2 and S1). We found that differences in costs between RA patients and matched controls with similar medical history appear years before the diagnosis of RA. Differences in cost increased from $56 per patient 8 years before diagnosis, rising to $277 per patient 2 years prior, and to $606 per patient 1 year before diagnosis. In the year of diagnosis, patients with RA cost $8,591 per patient meaning they were $4,141 per patient more expensive compared to patients matched on age, sex, and medical history who cost $4,449 per patient (Fig 3, Tables 2 and S2).

The top 5 cost categories for RA patients in the year of diagnosis (S3 Table) were inpatient costs ($1,746, 20.3%), physician fee for service payments to specialists ($952, 11.3%), outpatient care ($369, 4.3%), drug benefits ($317, 3.7%), and physician fee-for-service payments to general practitioners ($141, 1.6%).

### Trends in RA associated costs before and after diagnosis

In the year of diagnosis (Table 1), the RA associated costs were $4,142 per patient. The associated comorbidity costs of RA were $1,242 per patient and the underlying age-sex specific costs were $3,207 (Fig 3, Tables 2 and S1). Our findings show that by 8 years post diagnosis, the RA associated costs were $2,567 per patient, the RA comorbidity associated costs were $1,142 per patient and the age/sex underlying costs were $4,426 per patient (Tables 2 and S1). These results indicate that the RA associated costs are decreasing over time ($3088/$8163, 37.8%, at year 1 post diagnosis) compared to matched controls at 8 years post diagnosis ($2567/$8135, 31.5%, Fig 3, Tables 2 and S1). Over time, the RA comorbidity associated portion of costs remains almost constant from year 1 post diagnosis ($1263/$8163, 15.5%) to 8 years post diagnosis ($1142/$8135, 14.0%, Fig 3, Tables 2 and S1).

### Total and non-medication related health care costs grouped by calendar year of diagnosis (2002, 2009)

Costs were grouped by calendar year of RA diagnosis (2002, 2009) for total costs (Figs 4A and 5A., Table 3) and non-Medication costs (Figs 4B and 5B, Table 3). This analysis illustrates the association of calendar year of RA diagnosis on changes in total and non-medication related health care costs.

#### Total health care costs grouped by year of diagnosis

For patients diagnosed in 2002, the costs of RA patients were $6,870 per patient in the year of diagnosis increasing at 5 years post diagnosis to $8,095 per patient (Δ$1,225 per patient). Patients diagnosed in 2009 had a higher initial cost of treatment of $9,136 per patient but these costs decreased by $763 per patient at 5-years post diagnosis to $8,373 (Figs 4A and 5A, Tables 3 and S2).

#### Total non-medication related costs grouped by year of diagnosis

For patients diagnosed in 2002, the non-medication related costs of RA patients were $6,178 per patient in the year of diagnosis, increasing at 5 years post diagnosis to $6,735 per patient (Δ$557) (Fig 4B, Tables 3 and S2). Patients diagnosed in 2009 had higher initial non-medication related costs of $8,201 but these costs decreased by $1,761 at 5-years post diagnosis to $6,440(Figs 4B and 5B, Tables 3 and S2).

### Total publicly funded medication costs among RA patients

**Fig 6:**
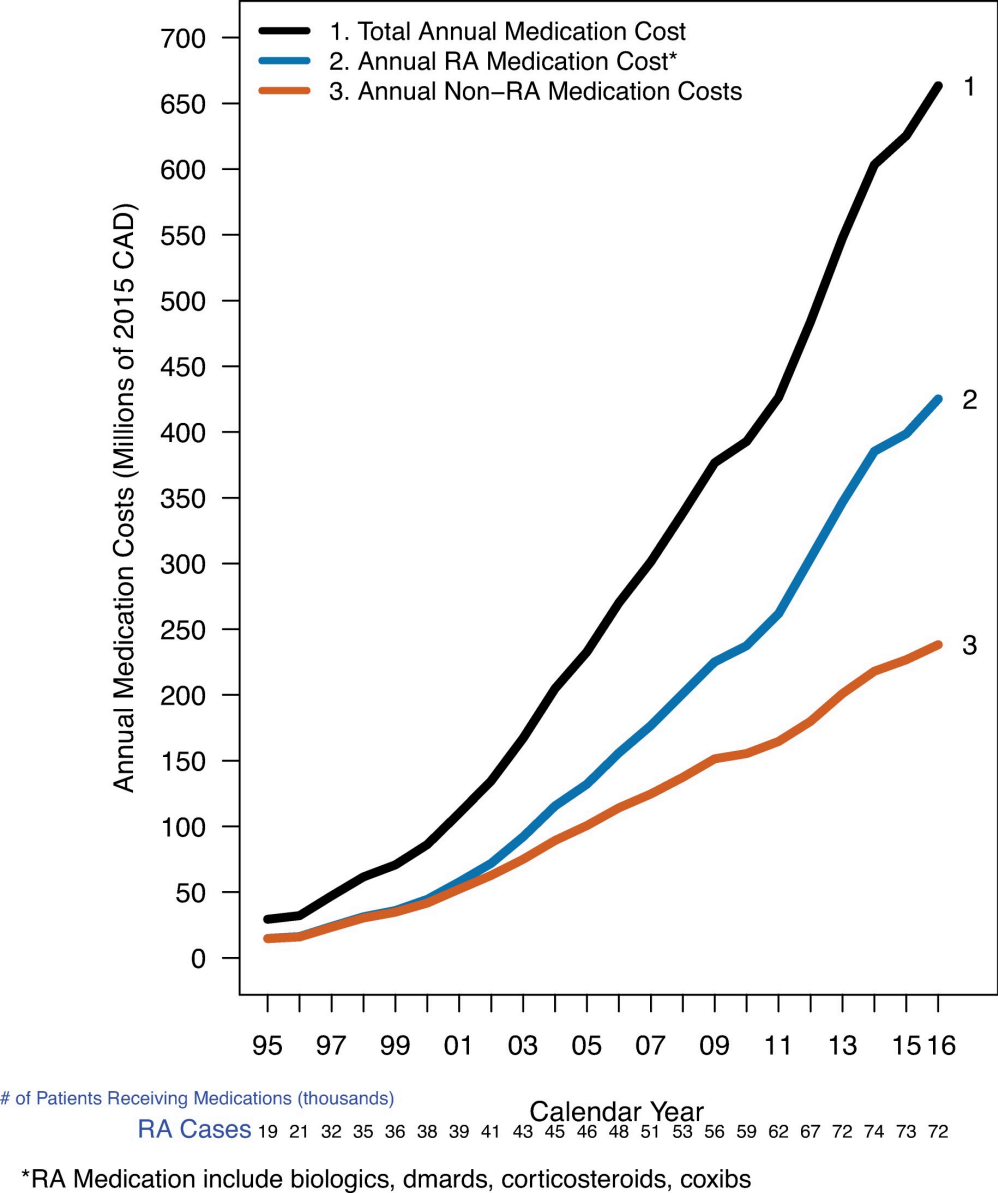
Total annual RA medication costs over time grouped by RA vs. Non−RA medications. X-axis: Calendar year, Y-axis: Annual Medication Costs (Millions of 2015 CAD), Grouping Variable: Medication Category).

**Fig 7:**
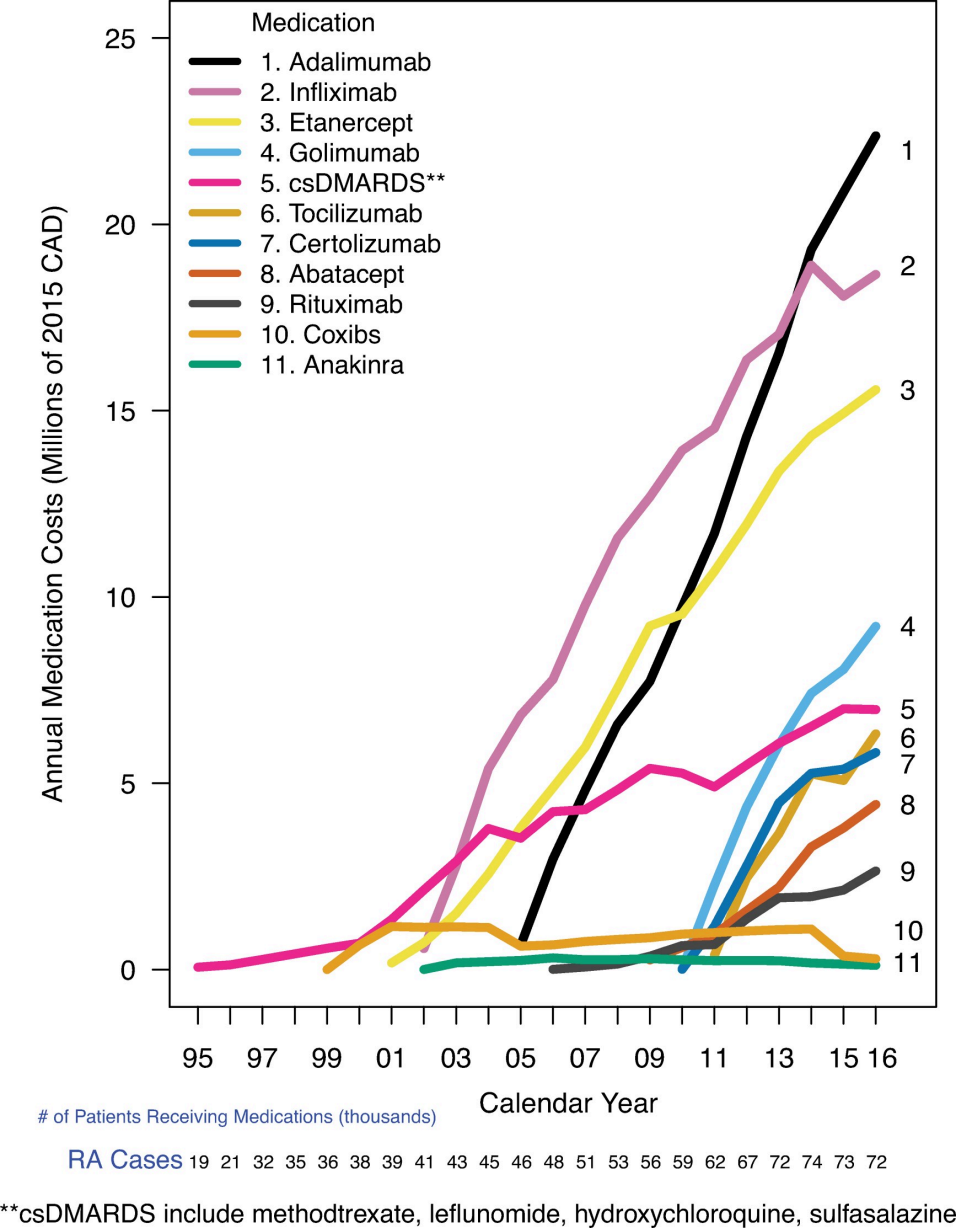
Annual RA medication costs over time. X-axis: Calendar year, Y-axis: Annual Medication Costs (Millions of 2015 CAD), Grouping Variable: Medication).

In 2016, 72,000 RA patients receiving publicly funded medications cost $663.4M, rising from $29.3M in 2001 (Fig 6). In 2001 50.3% ($14.8M) of all medication costs for RA patients were for RA-related medications rising to 64.0% ($425.2M/$663.4M) in 2016. When split by type of RA medication, (Fig 7) in 2016 the total cost of all 4 administered csDMARDs combined (methotrexate, leflunomide, sulfasalazine, hydroxychloroquine) was $7.0M (Fig 7) while the total costs of the top 5 biologics by total medication costs were Adalimumab ($22.4M), Infliximab ($18.7M), Etanercept ($15.6M), Golimumab ($9.2M), and Tocilizumab ($6.3M).

## Conclusions

We found that healthcare costs of RA patients were higher than costs for both age-sex matched, and age-sex-medical history matched controls. Increases in health care costs were detectable up to 15 years before the diagnosis of RA when compared to controls matched only on age and sex. Higher costs in RA patients were detectable up to 2-years prior to diagnosis when compared to patients matched on age-sex and the addition of medical history.

Our data showed that the per-patient costs of RA are the highest in the year of diagnosis and decline over time relative to matched controls. When separated by RA and comorbidity associated costs, at the year of disease diagnosis, a higher proportion of costs are associated with RA but this proportion decreases over time. In contrast, after diagnosis, the associated costs of RA related comorbidities remain constant as proportion of total cost.

After stratifying by calendar year of diagnosis to examine the association of temporal factors on health care costs (medical advances, clinical treatment advances, biological trends in disease severity) we found that patients diagnosed in 2002 years had greater decreases in cost between diagnosis and 5 years than those diagnosed in 2009, especially when medication costs were excluded. Because of the high cost of biologics, the interesting finding here is that the non-medication costs declined among patients diagnosed in later calendar years. Biologics can cost $15,000CAD/yr compared to about $900CAD/yr for csDMARDs, making comparisons between costs including medications unclear because of wide disparities in costs. we can infer if cost-deferral through improvements in treatments or care patterns are possible by measuring non-medication costs over time among patients with similar medical histories. For example, if non-medication related costs declined in later calendar years, adjusted for confounders, there is an underlying reason for cost reduction one of which could be differences in available pharmacologic treatment options.

We found that for patients diagnosed with RA, RA-related medication costs were about 50% of all medication costs paid by the public payor. For patients diagnosed with RA, Biologics were 4 of the top 5 medications having the highest total cost, with the combination of all 4 csDMARDs representing the 5^th^ most expensive RA related medication cost.

The main strengths of this study were the use of design techniques that minimized bias. The contribution of medical history to age-sex based matching improves upon study design methods relying on age-sex matching alone because controls are more similar to cases in the pre-diagnosis period. This similarity was used to separate the attributable costs of RA using statistical methods called Mahalanobis distance matrices instead of linear regression. Mahalanobis distance is a weighted measure of similarity between covariates that automatically upweights or downweighs variables based on the observed mean of a covariate in a patient compared to the shape of the variance of the population for that variable [34,39,42,43]. For uncorrelated data of a single covariate, regression and Mahalanobis distance will perform similarly. However, when data is highly correlated which is present in patients with chronic diseases like rheumatoid arthritis, we see large differences between Mahalanobis Distances and unweighted statistical methods like regression. Our assumption that medical histories contain correlated data are shown by the large difference in medical histories between patients with RA and matched controls. On the macro scale over thousands of covariates, Mahalanobis Distance ensures that patients are similar in the proportion and shape of the variance of all comorbidities. Mean values that deviate in the same direction of the variance are downweighed while mean values that are deviating in opposite directions to the sample (i.e. outliers) are up-weighted. Linear regression is unable to reliably concurrently adjust for hundreds of covariates over time. We can see from Fig 4. the difference between the matched controls on pre-diagnosis medical histories (before 0 on the x-axis) and the patients matched on age and sex are large, indicating the considerable benefit of adding a distance matrix match to age and sex matching alone. Numerically, we see that patients with a Mahalanobis distance matrix match are more similar on each clinical covariate by at least 30% (S3 Table).

Our study further improved on existing costing studies considering costs in relation to disease diagnosis and calendar year instead of calendar year alone. The advantage of this design is that we ensured that cases and controls were matched at identical calendar time periods to reduce bias due to changing cost patterns over time and changing cost measurement categories. In health care systems, changing cost measurements are inevitable as a result of changes over time in health care delivery, technologies, and preferences. Therefore, measurement of longitudinal costs over a long-term time horizon will have changing availability of costs and differences in which costs are included in each category (S2 File). Many costing studies ignore this issue creating immortal time bias [44,45], but our use of matched case control design on year of birth and calendar year ensured that comparisons were made between patients in the same time period for which costing measurement was identical in both cases and controls. We present our findings always as a comparison difference between cases and controls to highlight that the difference in cost in each time period is the meaningful value for researchers and decision makers to interpret because of the identical collection of available costs. When our results are presented between time periods, we show the difference between matched cases and controls which is robust to changes in costing measurement over time.

Our study limitations are common to all matching studies: potential missing unobserved covariates that could contribute to differences between the case and control groups. This concern could be improved by the addition of clinical chart data from electronic medical records to adjust for clinical differences between the cases and matched controls; however, these data were not available. Other limitations were that full medication costs were captured for patients above 65 with uncaptured data for privately paid for medication costs in the under-65 population. We addressed this concern by matching patients based on common time periods which ensures no immortal measurement bias occurs. Finally, costing data on lost work productivity, and non-health care related costs including caregiver time could be significant unmeasured costs from the societal perspective. The population under study was restricted to adult-onset RA only because of the significantly different and heterogenous clinical phenotype of Juvenile Idiopathic Arthritis (JIA) combined with the lack of a validated administrative algorithm for detecting these patients. There is work underway to validate an administrative algorithm for patients under the age of 16 with Juvenile Idiopathic Arthritis (JIA) and the authors expect a separate costing analysis for this patient population. Because the adult and juvenile populations have clinically meaningful differences, combining the two populations in a costing study would likely be suboptimal from a health services research measurement perspective.

Comparisons of costing studies, either comparisons across countries or within countries are difficult because of the use divergent methods to measure costs, the scope of costs captured, and differences in cohort ascertainment methods reflect local differences in health care systems [10,11,13,15,46–53]. Our study is most comparable to results from Sweden [10], where a similar single payer system yielded similar trends in costs detectable up to 1 year before diagnosis and costs decreasing after 1-year post diagnosis [10]. The costs of newly diagnosed RA in Sweden were $9,239 CAD per patient on average, compared to $8,591 CAD per patient in our study.

This study showed the health care cost implications for RA treatment by clinicians. Up to the year of diagnosis we observed rises in health care costs with declining costs in the post-diagnosis years. For clinicians, the data could be interpreted as treatment benefit, strengthening the argument of further resources allocated to early detection and rapid treatment.

Future research could target critical periods as cost avoidance strategies including early detection of disease onset. These findings show that reductions in RA related costs are measurable after diagnosis with more detailed data and methods separating the causal relationships between occurrence of disease and health care costs. As treatment strategies improve over time, these improvements can change the amount and distribution of health care costs.

## Supporting information

S1 Fig Annual per-patient non-medication health care costs over time grouped by diagnosis year (all diagnosis years) before and after rheumatoid arthritis diagnosis (public payer’s perspective). (DOCX)

S1 Table Patient outcomes by cost category at year of diagnosis and 1, 2, 5, and 8-Year pre-and post-diagnosis (data source for Fig 3). (DOCX)

S2 Table Patient outcomes by year of exposure and cost category. (DOCX)

S3 Table Demographics and costs by exposure group and year. (DOCX)

S1 File Matching variables and description of Hopkins MEDC. (DOCX)

S2 File Costing categories and timeline data (and price) availability for cost calculation. (DOCX)
