# Supplementary material for: Health care costs of rheumatoid arthritis: A longitudinal population study
Source: PLoS One. 2021 May 6;16(5):e0251334. doi: 10.1371/journal.pone.0251334 (PMC8101709; doi:10.1371/journal.pone.0251334)
Supplement: S1 Fig — (DOCX) [file pone.0251334.s001.docx]

Supplement 6: Annual Per-Patient non-Medication Health Care Costs Over Time Grouped by Diagnosis Year (All Diagnosis Years) Before and After Rheumatoid Arthritis Diagnosis (Public Payer’s Perspective)

*Matched on 27 Major Expanded Diagnosis Categories (See Supplement 7 for Matching Variables)
