# Supplementary material for: Health care costs of rheumatoid arthritis: A longitudinal population study
Source: PLoS One. 2021 May 6;16(5):e0251334. doi: 10.1371/journal.pone.0251334 (PMC8101709; doi:10.1371/journal.pone.0251334)
Supplement: S1 Table — (DOCX) [file pone.0251334.s002.docx]

| Years Before / After Diagnosis | RA Cases (2015 CAD/patient) | Age/Sex/Medical History Matched Controls (2015 CAD/patient) | Average Difference | Age/Sex Matched Controls (2015 CAD/patient) | Average Difference (2015 CAD/patient) | Direct RA Associated Costs (2015 CAD/patient) | Indirect RA Associated (2015 CAD/patient) | Age/Sex Related Costs (2015 CAD/patient) |
| --- | --- | --- | --- | --- | --- | --- | --- | --- |
| (n pairs of cases and controls) |  |  | (2015 CAD/patient) |  |  |  |  |  |
| Inpatient Care |  |  |  |  |  |  |  |  |
| -8 | 295 | 263 | 32 | 181 | 114 | 32 | 82 | 181 |
| -5 | 415 | 409 | 6 | 273 | 142 | 6 | 136 | 273 |
| -2 | 736 | 675 | 61 | 460 | 276 | 61 | 215 | 460 |
| -1 | 962 | 787 | 175 | 538 | 424 | 175 | 249 | 538 |
| 0 | 2672 | 926 | 1746 | 651 | 2021 | 1746 | 275 | 651 |
| 1 | 2164 | 1180 | 984 | 888 | 1276 | 984 | 292 | 888 |
| 2 | 1956 | 1180 | 776 | 911 | 1045 | 776 | 269 | 911 |
| 5 | 1859 | 1165 | 694 | 982 | 877 | 694 | 183 | 982 |
| 8 | 1857 | 1250 | 607 | 995 | 862 | 607 | 255 | 995 |
| Physician Fee for Service Billings (Specialist) | RA Cases | Age/Sex/Medical History Matched Controls | Average Difference | Age/Sex Matched Controls | Average Difference | Direct RA Associated Costs | Indirect RA Associated | Age/Sex Related Costs |
| -8 | 569 | 535 | 34 | 368 | 201 | 34 | 167 | 368 |
| -5 | 655 | 604 | 51 | 415 | 240 | 51 | 189 | 415 |
| -2 | 803 | 657 | 146 | 471 | 332 | 146 | 186 | 471 |
| -1 | 927 | 679 | 248 | 489 | 438 | 248 | 190 | 489 |
| 0 | 1656 | 704 | 952 | 509 | 1147 | 952 | 195 | 509 |
| 1 | 1360 | 727 | 633 | 538 | 822 | 633 | 189 | 538 |
| 2 | 1234 | 716 | 518 | 544 | 690 | 518 | 172 | 544 |
| 5 | 1102 | 707 | 395 | 554 | 548 | 395 | 153 | 554 |
| 8 | 1029 | 702 | 327 | 549 | 480 | 327 | 153 | 549 |
| Drug Benefits | RA Cases | Age/Sex/Medical History Matched Controls | Average Difference | Age/Sex Matched Controls | Average Difference | Direct RA Associated Costs | Indirect RA Associated | Age/Sex Related Costs |
| -8 | 256 | 256 | 0 | 182 | 74 | 0 | 74 | 182 |
| -5 | 382 | 365 | 17 | 260 | 122 | 17 | 105 | 260 |
| -2 | 589 | 515 | 74 | 378 | 211 | 74 | 137 | 378 |
| -1 | 696 | 575 | 121 | 421 | 275 | 121 | 154 | 421 |
| 0 | 958 | 641 | 317 | 475 | 483 | 317 | 166 | 475 |
| 1 | 1242 | 687 | 555 | 520 | 722 | 555 | 167 | 520 |
| 2 | 1415 | 721 | 694 | 542 | 873 | 694 | 179 | 542 |
| 5 | 1658 | 734 | 924 | 578 | 1080 | 924 | 156 | 578 |
| 8 | 1751 | 741 | 1010 | 611 | 1140 | 1010 | 130 | 611 |
| Outpatient | RA Cases | Age/Sex/Medical History Matched Controls | Average Difference | Age/Sex Matched Controls | Average Difference | Direct RA Associated Costs | Indirect RA Associated | Age/Sex Related Costs |
| -8 | 67 | 61 | 6 | 43 | 24 | 6 | 18 | 43 |
| -5 | 146 | 122 | 24 | 89 | 57 | 24 | 33 | 89 |
| -2 | 268 | 195 | 73 | 140 | 128 | 73 | 55 | 140 |
| -1 | 333 | 217 | 116 | 161 | 172 | 116 | 56 | 161 |
| 0 | 618 | 249 | 369 | 183 | 435 | 369 | 66 | 183 |
| 1 | 598 | 278 | 320 | 205 | 393 | 320 | 73 | 205 |
| 2 | 564 | 293 | 271 | 217 | 347 | 271 | 76 | 217 |
| 5 | 581 | 346 | 235 | 267 | 314 | 235 | 79 | 267 |
| 8 | 519 | 329 | 190 | 259 | 260 | 190 | 70 | 259 |
| Physician (General Practitioner) | RA Cases | Age/Sex/Medical History Matched Controls | Average Difference | Age/Sex Matched Controls | Average Difference | Direct RA Associated Costs | Indirect RA Associated | Age/Sex Related Costs |
| -8 | 279 | 288 | -9 | 201 | 78 | -9 | 87 | 201 |
| -5 | 281 | 288 | -7 | 200 | 81 | -7 | 88 | 200 |
| -2 | 285 | 271 | 14 | 200 | 85 | 14 | 71 | 200 |
| -1 | 305 | 275 | 30 | 196 | 109 | 30 | 79 | 196 |
| 0 | 414 | 273 | 141 | 194 | 220 | 141 | 79 | 194 |
| 1 | 352 | 272 | 80 | 200 | 152 | 80 | 72 | 200 |
| 2 | 322 | 266 | 56 | 195 | 127 | 56 | 71 | 195 |
| 5 | 285 | 250 | 35 | 187 | 98 | 35 | 63 | 187 |
| 8 | 255 | 221 | 34 | 173 | 82 | 34 | 48 | 173 |
| Laboratory Costs | RA Cases | Age/Sex/Medical History Matched Controls | Average Difference | Age/Sex Matched Controls | Average Difference | Direct RA Associated Costs | Indirect RA Associated | Age/Sex Related Costs |
| -8 | 112 | 107 | 5 | 79 | 33 | 5 | 28 | 79 |
| -5 | 124 | 114 | 10 | 84 | 40 | 10 | 30 | 84 |
| -2 | 141 | 115 | 26 | 86 | 55 | 26 | 29 | 86 |
| -1 | 163 | 116 | 47 | 87 | 76 | 47 | 29 | 87 |
| 0 | 307 | 118 | 189 | 89 | 218 | 189 | 29 | 89 |
| 1 | 256 | 117 | 139 | 90 | 166 | 139 | 27 | 90 |
| 2 | 228 | 115 | 113 | 90 | 138 | 113 | 25 | 90 |
| 5 | 188 | 110 | 78 | 89 | 99 | 78 | 21 | 89 |
| 8 | 166 | 104 | 62 | 86 | 80 | 62 | 18 | 86 |
| Home Care | RA Cases | Age/Sex/Medical History Matched Controls | Average Difference | Age/Sex Matched Controls | Average Difference | Direct RA Associated Costs | Indirect RA Associated | Age/Sex Related Costs |
| -8 | 39 | 43 | -4 | 28 | 11 | -4 | 15 | 28 |
| -5 | 76 | 79 | -3 | 52 | 24 | -3 | 27 | 52 |
| -2 | 157 | 151 | 6 | 104 | 53 | 6 | 47 | 104 |
| -1 | 221 | 188 | 33 | 129 | 92 | 33 | 59 | 129 |
| 0 | 410 | 237 | 173 | 168 | 242 | 173 | 69 | 168 |
| 1 | 473 | 288 | 185 | 211 | 262 | 185 | 77 | 211 |
| 2 | 480 | 303 | 177 | 225 | 255 | 177 | 78 | 225 |
| 5 | 502 | 323 | 179 | 252 | 250 | 179 | 71 | 252 |
| 8 | 507 | 352 | 155 | 286 | 221 | 155 | 66 | 286 |
| Rehabilitation | RA Cases | Age/Sex/Medical History Matched Controls | Average Difference | Age/Sex Matched Controls | Average Difference | Direct RA Associated Costs | Indirect RA Associated | Age/Sex Related Costs |
| -8 | 22 | 18 | 4 | 16 | 6 | 4 | 2 | 16 |
| -5 | 32 | 33 | -1 | 22 | 10 | -1 | 11 | 22 |
| -2 | 60 | 55 | 5 | 41 | 19 | 5 | 14 | 41 |
| -1 | 82 | 63 | 19 | 51 | 31 | 19 | 12 | 51 |
| 0 | 297 | 81 | 216 | 59 | 238 | 216 | 22 | 59 |
| 1 | 214 | 86 | 128 | 76 | 138 | 128 | 10 | 76 |
| 2 | 190 | 98 | 92 | 77 | 113 | 92 | 21 | 77 |
| 5 | 189 | 94 | 95 | 83 | 106 | 95 | 11 | 83 |
| 8 | 164 | 95 | 69 | 89 | 75 | 69 | 6 | 89 |
| Emergency Department | RA Cases | Age/Sex/Medical History Matched Controls | Average Difference | Age/Sex Matched Controls | Average Difference | Direct RA Associated Costs | Indirect RA Associated | Age/Sex Related Costs |
| -8 | 49 | 46 | 3 | 30 | 19 | 3 | 16 | 30 |
| -5 | 77 | 69 | 8 | 46 | 31 | 8 | 23 | 46 |
| -2 | 123 | 103 | 20 | 69 | 54 | 20 | 34 | 69 |
| -1 | 157 | 118 | 39 | 79 | 78 | 39 | 39 | 79 |
| 0 | 271 | 135 | 136 | 90 | 181 | 136 | 45 | 90 |
| 1 | 216 | 148 | 68 | 107 | 109 | 68 | 41 | 107 |
| 2 | 219 | 159 | 60 | 115 | 104 | 60 | 44 | 115 |
| 5 | 210 | 161 | 49 | 119 | 91 | 49 | 42 | 119 |
| 8 | 203 | 161 | 42 | 122 | 81 | 42 | 39 | 122 |
| Same Day Surgery | RA Cases | Age/Sex/Medical History Matched Controls | Average Difference | Age/Sex Matched Controls | Average Difference | Direct RA Associated Costs | Indirect RA Associated | Age/Sex Related Costs |
| -8 | 59 | 50 | 9 | 40 | 19 | 9 | 10 | 40 |
| -5 | 95 | 81 | 14 | 59 | 36 | 14 | 22 | 59 |
| -2 | 146 | 115 | 31 | 87 | 59 | 31 | 28 | 87 |
| -1 | 168 | 130 | 38 | 95 | 73 | 38 | 35 | 95 |
| 0 | 202 | 141 | 61 | 107 | 95 | 61 | 34 | 107 |
| 1 | 205 | 149 | 56 | 115 | 90 | 56 | 34 | 115 |
| 2 | 210 | 158 | 52 | 122 | 88 | 52 | 36 | 122 |
| 5 | 197 | 156 | 41 | 122 | 75 | 41 | 34 | 122 |
| 8 | 182 | 153 | 29 | 117 | 65 | 29 | 36 | 117 |
| Continuing Care | RA Cases | Age/Sex/Medical History Matched Controls | Average Difference | Age/Sex Matched Controls | Average Difference | Direct RA Associated Costs | Indirect RA Associated | Age/Sex Related Costs |
| -8 | 1 | 9 | -8 | 8 | -7 | -8 | 1 | 8 |
| -5 | 8 | 30 | -22 | 12 | -4 | -22 | 18 | 12 |
| -2 | 27 | 61 | -34 | 37 | -10 | -34 | 24 | 37 |
| -1 | 48 | 98 | -50 | 54 | -6 | -50 | 44 | 54 |
| 0 | 171 | 143 | 28 | 74 | 97 | 28 | 69 | 74 |
| 1 | 243 | 167 | 76 | 126 | 117 | 76 | 41 | 126 |
| 2 | 221 | 168 | 53 | 136 | 85 | 53 | 32 | 136 |
| 5 | 195 | 174 | 21 | 135 | 60 | 21 | 39 | 135 |
| 8 | 253 | 207 | 46 | 123 | 130 | 46 | 84 | 123 |
| Long-Term Care | RA Cases | Age/Sex/Medical History Matched Controls | Average Difference | Age/Sex Matched Controls | Average Difference | Direct RA Associated Costs | Indirect RA Associated | Age/Sex Related Costs |
| -8 | 3 | 18 | -15 | 13 | -10 | -15 | 5 | 13 |
| -5 | 13 | 58 | -45 | 40 | -27 | -45 | 18 | 40 |
| -2 | 43 | 170 | -127 | 144 | -101 | -127 | 26 | 144 |
| -1 | 64 | 257 | -193 | 205 | -141 | -193 | 52 | 205 |
| 0 | 157 | 370 | -213 | 295 | -138 | -213 | 75 | 295 |
| 1 | 340 | 499 | -159 | 387 | -47 | -159 | 112 | 387 |
| 2 | 409 | 538 | -129 | 405 | 4 | -129 | 133 | 405 |
| 5 | 524 | 629 | -105 | 497 | 27 | -105 | 132 | 497 |
| 8 | 618 | 647 | -29 | 557 | 61 | -29 | 90 | 557 |
| Capitation | RA Cases | Age/Sex/Medical History Matched Controls | Average Difference | Age/Sex Matched Controls | Average Difference | Direct RA Associated Costs | Indirect RA Associated | Age/Sex Related Costs |
| -8 | 13 | 11 | 2 | 10 | 3 | 2 | 1 | 10 |
| -5 | 39 | 33 | 6 | 31 | 8 | 6 | 2 | 31 |
| -2 | 74 | 65 | 9 | 59 | 15 | 9 | 6 | 59 |
| -1 | 87 | 76 | 11 | 69 | 18 | 11 | 7 | 69 |
| 0 | 100 | 87 | 13 | 79 | 21 | 13 | 8 | 79 |
| 1 | 108 | 94 | 14 | 85 | 23 | 14 | 9 | 85 |
| 2 | 114 | 99 | 15 | 90 | 24 | 15 | 9 | 90 |
| 5 | 131 | 120 | 11 | 109 | 22 | 11 | 11 | 109 |
| 8 | 143 | 136 | 7 | 125 | 18 | 12 | 11 | 125 |
| Cancer Clinics | RA Cases | Age/Sex/Medical History Matched Controls | Average Difference | Age/Sex Matched Controls | Average Difference | Direct RA Associated Costs | Indirect RA Associated | Age/Sex Related Costs |
| -8 | 9 | 6 | 3 | 8 | 1 | 3 | -2 | 8 |
| -5 | 18 | 20 | -2 | 16 | 2 | -2 | 4 | 16 |
| -2 | 40 | 46 | -6 | 40 | 0 | -6 | 6 | 40 |
| -1 | 59 | 57 | 2 | 54 | 5 | 2 | 3 | 54 |
| 0 | 83 | 80 | 3 | 75 | 8 | 3 | 5 | 75 |
| 1 | 96 | 94 | 2 | 83 | 13 | 2 | 11 | 83 |
| 2 | 114 | 97 | 17 | 86 | 28 | 17 | 11 | 86 |
| 5 | 131 | 121 | 10 | 103 | 28 | 10 | 18 | 103 |
| 8 | 137 | 116 | 21 | 113 | 24 | 21 | 3 | 113 |
| Dialysis Clinics | RA Cases | Age/Sex/Medical History Matched Controls | Average Difference | Age/Sex Matched Controls | Average Difference | Direct RA Associated Costs | Indirect RA Associated | Age/Sex Related Costs |
| -8 | 5 | 8 | -3 | 3 | 2 | -3 | 5 | 3 |
| -5 | 17 | 19 | -2 | 13 | 4 | -2 | 6 | 13 |
| -2 | 46 | 42 | 4 | 27 | 19 | 4 | 15 | 27 |
| -1 | 57 | 65 | -8 | 33 | 24 | -8 | 32 | 33 |
| 0 | 83 | 92 | -9 | 47 | 36 | -9 | 45 | 47 |
| 1 | 104 | 99 | 5 | 61 | 43 | 5 | 38 | 61 |
| 2 | 104 | 97 | 7 | 62 | 42 | 7 | 35 | 62 |
| 5 | 141 | 137 | 4 | 85 | 56 | 4 | 52 | 85 |
| 8 | 146 | 128 | 18 | 72 | 74 | 18 | 56 | 72 |
| Mental Health Inpatient | RA Cases | Age/Sex/Medical History Matched Controls | Average Difference | Age/Sex Matched Controls | Average Difference | Direct RA Associated Costs | Indirect RA Associated | Age/Sex Related Costs |
| -8 | 8 | 16 | -8 | 5 | 3 | -8 | 11 | 5 |
| -5 | 14 | 48 | -34 | 29 | -15 | -34 | 19 | 29 |
| -2 | 35 | 68 | -33 | 33 | 2 | -33 | 35 | 33 |
| -1 | 35 | 73 | -38 | 34 | 1 | -38 | 39 | 34 |
| 0 | 50 | 86 | -36 | 44 | 6 | -36 | 42 | 44 |
| 1 | 56 | 97 | -41 | 47 | 9 | -41 | 50 | 47 |
| 2 | 64 | 90 | -26 | 61 | 3 | -26 | 29 | 61 |
| 5 | 78 | 116 | -38 | 68 | 10 | -38 | 48 | 68 |
| 8 | 94 | 132 | -38 | 65 | 29 | -38 | 67 | 65 |
| Assistive Devices | RA Cases | Age/Sex/Medical History Matched Controls | Average Difference | Age/Sex Matched Controls | Average Difference | Direct RA Associated Costs | Indirect RA Associated | Age/Sex Related Costs |
| -8 | 3 | 4 | -1 | 2 | 1 | -1 | 2 | 2 |
| -5 | 7 | 7 | 0 | 4 | 3 | 0 | 3 | 4 |
| -2 | 8 | 8 | 0 | 5 | 3 | 0 | 3 | 5 |
| -1 | 10 | 9 | 1 | 7 | 3 | 1 | 2 | 7 |
| 0 | 15 | 10 | 5 | 7 | 8 | 5 | 3 | 7 |
| 1 | 19 | 11 | 8 | 7 | 12 | 8 | 4 | 7 |
| 2 | 18 | 14 | 4 | 9 | 9 | 4 | 5 | 9 |
| 5 | 16 | 13 | 3 | 9 | 7 | 3 | 4 | 9 |
| 8 | 6 | 5 | 1 | 4 | 2 | 1 | 1 | 4 |
