# Supplementary material for: Health care costs of rheumatoid arthritis: A longitudinal population study
Source: PLoS One. 2021 May 6;16(5):e0251334. doi: 10.1371/journal.pone.0251334 (PMC8101709; doi:10.1371/journal.pone.0251334)
Supplement: S2 Table — (DOCX) [file pone.0251334.s003.docx]

Supplement 4: Patient Outcomes by Year of Exposure and Cost Category

| Incident Year | Years Before / After RA Diagnosis (n pairs), n biologics | RA Cases  (2015 CAD/patient) | Age/Sex/Disease Matched Controls (2015 CAD/patient) | Average Difference (2015 CAD/patient) | Age/Sex Matched Controls (2015 CAD/patient) | Average Difference (2015 CAD/patient) | Direct RA Associated Costs | Indirect RA Associated | Age/Sex Related Costs |
| --- | --- | --- | --- | --- | --- | --- | --- | --- | --- |
| Cost Category | **Inpatient Cost** | | | | | | | | |
| 2002 | -5 (n=5,322), 0 | 0 | 0 | 0 | 0 | 0 | 0 | 0 | 0 |
|  | -2 (n=5,322), 0 | 2 | 8 | -7 | 3 | -1 | -7 | 5 | 3 |
|  | -1 (n=5,322), 0 | 76 | 99 | -23 | 46 | 30 | -23 | 53 | 46 |
|  | 0 (n=5,322), <8 | 2722 | 940 | 1782 | 538 | 2185 | 1782 | 402 | 538 |
|  | 1 (n=5,322), 11 | 2043 | 1209 | 834 | 954 | 1088 | 834 | 255 | 954 |
|  | 2 (n=5,322), 24 | 1999 | 1309 | 691 | 905 | 1094 | 691 | 404 | 905 |
|  | 5 (n=5,322), 31 | 1958 | 1263 | 695 | 897 | 1061 | 695 | 366 | 897 |
|  | 8 (n=5,322), 27 | 1959 | 1174 | 785 | 984 | 975 | 785 | 190 | 984 |
| 2009 | -8 (n=6637) | 12 | 36 | -23 | 29 | -17 | -23 | 7 | 29 |
|  | -5 (n=6637), <8 | 672 | 678 | -6 | 471 | 201 | -6 | 207 | 471 |
|  | -2 (n=6637), <8 | 875 | 740 | 135 | 568 | 307 | 135 | 172 | 568 |
|  | -1 (n=6637), 8 | 1181 | 768 | 413 | 654 | 527 | 413 | 114 | 654 |
|  | 0 (n=6637), 20 | 2912 | 882 | 2030 | 774 | 2138 | 2030 | 108 | 774 |
|  | 1 (n=6637), 45 | 2232 | 1198 | 1034 | 906 | 1326 | 1034 | 292 | 906 |
|  | 2 (n=6637), 91 | 1890 | 1131 | 759 | 1029 | 861 | 759 | 102 | 1029 |
|  | 5 (n=6637), 66 | 1953 | 1207 | 746 | 1025 | 927 | 746 | 182 | 1025 |
| Cost Category | Physician Fee for Service Billings (Specialist) | | | | | | | | |
| 2002 | -5 (n=5,322), 0 | 681 | 693 | -13 | 442 | 238 | -13 | 251 | 442 |
|  | -2 (n=5,322), 0 | 753 | 708 | 45 | 442 | 311 | 45 | 266 | 442 |
|  | -1 (n=5,322), 0 | 883 | 722 | 161 | 466 | 417 | 161 | 256 | 466 |
|  | 0 (n=5,322), <8 | 1529 | 731 | 798 | 477 | 1052 | 798 | 254 | 477 |
|  | 1 (n=5,322), 11 | 1149 | 695 | 454 | 497 | 653 | 454 | 198 | 497 |
|  | 2 (n=5,322), 24 | 1093 | 698 | 395 | 499 | 594 | 395 | 199 | 499 |
|  | 5 (n=5,322), 31 | 1032 | 692 | 340 | 479 | 554 | 340 | 213 | 479 |
|  | 8 (n=5,322), 27 | 1030 | 718 | 312 | 529 | 500 | 312 | 189 | 529 |
| 2009 | -8 (n=6637) | 576 | 539 | 38 | 372 | 204 | 38 | 167 | 372 |
|  | -5 (n=6637), <8 | 626 | 572 | 54 | 404 | 222 | 54 | 168 | 404 |
|  | -2 (n=6637), <8 | 780 | 618 | 161 | 460 | 320 | 161 | 158 | 460 |
|  | -1 (n=6637), 8 | 928 | 656 | 272 | 506 | 422 | 272 | 150 | 506 |
|  | 0 (n=6637), 20 | 1679 | 704 | 975 | 540 | 1139 | 975 | 164 | 540 |
|  | 1 (n=6637), 45 | 1457 | 783 | 674 | 581 | 875 | 674 | 202 | 581 |
|  | 2 (n=6637), 91 | 1378 | 751 | 627 | 616 | 762 | 627 | 135 | 616 |
|  | 5 (n=6637), 66 | 1168 | 710 | 458 | 555 | 612 | 458 | 155 | 555 |
| Cost Category | Drug Benefits | | | | | | | | |
| 2002 | -5 (n=5,322), 0 | 216 | 234 | -17 | 158 | 58 | -17 | 76 | 158 |
|  | -2 (n=5,322), 0 | 383 | 391 | -8 | 257 | 126 | -8 | 134 | 257 |
|  | -1 (n=5,322), 0 | 477 | 451 | 26 | 313 | 164 | 26 | 138 | 313 |
|  | 0 (n=5,322), <8 | 691 | 545 | 147 | 376 | 315 | 147 | 169 | 376 |
|  | 1 (n=5,322), 11 | 932 | 621 | 310 | 432 | 500 | 310 | 189 | 432 |
|  | 2 (n=5,322), 24 | 1115 | 676 | 440 | 475 | 640 | 440 | 201 | 475 |
|  | 5 (n=5,322), 31 | 1362 | 762 | 600 | 557 | 805 | 600 | 205 | 557 |
|  | 8 (n=5,322), 27 | 1496 | 787 | 709 | 595 | 901 | 709 | 192 | 595 |
| 2009 | -8 (n=6637) | 235 | 234 | 1 | 175 | 60 | 1 | 59 | 175 |
|  | -5 (n=6637), <8 | 434 | 387 | 46 | 286 | 148 | 46 | 101 | 286 |
|  | -2 (n=6637), <8 | 629 | 546 | 83 | 416 | 214 | 83 | 130 | 416 |
|  | -1 (n=6637), 8 | 714 | 603 | 111 | 468 | 246 | 111 | 135 | 468 |
|  | 0 (n=6637), 20 | 936 | 677 | 259 | 529 | 407 | 259 | 148 | 529 |
|  | 1 (n=6637), 45 | 1133 | 679 | 454 | 532 | 602 | 454 | 147 | 532 |
|  | 2 (n=6637), 91 | 1340 | 684 | 655 | 501 | 839 | 655 | 183 | 501 |
|  | 5 (n=6637), 66 | 1933 | 750 | 1183 | 619 | 1315 | 1183 | 131 | 619 |
| Cost Category | Outpatient | | | | | | | | |
| 2002 | -5 (n=5,322), 0 | 0 | 0 | 0 | 0 | 0 | 0 | 0 | 0 |
|  | -2 (n=5,322), 0 | 0 | 0 | 0 | 0 | 0 | 0 | 0 | 0 |
|  | -1 (n=5,322), 0 | 0 | 0 | 0 | 0 | 0 | 0 | 0 | 0 |
|  | 0 (n=5,322), <8 | 0 | 0 | 0 | 0 | 0 | 0 | 0 | 0 |
|  | 1 (n=5,322), 11 | 0 | 0 | 0 | 0 | 0 | 0 | 0 | 0 |
|  | 2 (n=5,322), 24 | 0 | 0 | 0 | 0 | 0 | 0 | 0 | 0 |
|  | 5 (n=5,322), 31 | 671 | 426 | 245 | 290 | 381 | 245 | 136 | 290 |
|  | 8 (n=5,322), 27 | 562 | 383 | 178 | 274 | 288 | 178 | 109 | 274 |
| 2009 | -8 (n=6637) | 0 | 0 | 0 | 0 | 0 | 0 | 0 | 0 |
|  | -5 (n=6637), <8 | 0 | 0 | 0 | 0 | 0 | 0 | 0 | 0 |
|  | -2 (n=6637), <8 | 458 | 358 | 100 | 257 | 201 | 100 | 101 | 257 |
|  | -1 (n=6637), 8 | 514 | 364 | 150 | 272 | 242 | 150 | 92 | 272 |
|  | 0 (n=6637), 20 | 812 | 363 | 449 | 296 | 516 | 449 | 67 | 296 |
|  | 1 (n=6637), 45 | 831 | 380 | 450 | 279 | 551 | 450 | 101 | 279 |
|  | 2 (n=6637), 91 | 711 | 340 | 371 | 258 | 453 | 371 | 82 | 258 |
|  | 5 (n=6637), 66 | 564 | 315 | 249 | 256 | 308 | 249 | 59 | 256 |
| Cost Category | Physician (General Practitioner) | | | | | | | | |
| 2002 | -5 (n=5,322), 0 | 387 | 400 | -13 | 261 | 127 | -13 | 139 | 261 |
|  | -2 (n=5,322), 0 | 366 | 378 | -12 | 244 | 121 | -12 | 134 | 244 |
|  | -1 (n=5,322), 0 | 398 | 375 | 23 | 245 | 153 | 23 | 130 | 245 |
|  | 0 (n=5,322), <8 | 507 | 364 | 143 | 255 | 253 | 143 | 109 | 255 |
|  | 1 (n=5,322), 11 | 423 | 350 | 73 | 248 | 175 | 73 | 102 | 248 |
|  | 2 (n=5,322), 24 | 399 | 339 | 60 | 245 | 154 | 60 | 94 | 245 |
|  | 5 (n=5,322), 31 | 347 | 285 | 62 | 219 | 129 | 62 | 66 | 219 |
|  | 8 (n=5,322), 27 | 282 | 244 | 37 | 182 | 99 | 37 | 62 | 182 |
| 2009 | -8 (n=6637) | 293 | 298 | -6 | 221 | 71 | -6 | 77 | 221 |
|  | -5 (n=6637), <8 | 283 | 278 | 5 | 224 | 58 | 5 | 54 | 224 |
|  | -2 (n=6637), <8 | 298 | 270 | 29 | 242 | 57 | 29 | 28 | 242 |
|  | -1 (n=6637), 8 | 315 | 273 | 42 | 235 | 80 | 42 | 38 | 235 |
|  | 0 (n=6637), 20 | 400 | 263 | 136 | 197 | 202 | 136 | 66 | 197 |
|  | 1 (n=6637), 45 | 347 | 249 | 98 | 197 | 150 | 98 | 52 | 197 |
|  | 2 (n=6637), 91 | 308 | 251 | 57 | 186 | 121 | 57 | 65 | 186 |
|  | 5 (n=6637), 66 | 254 | 207 | 46 | 166 | 87 | 46 | 41 | 166 |
| Cost Category | Laboratory Costs | | | | | | | | |
| 2002 | -5 (n=5,322), 0 | 153 | 131 | 21 | 98 | 55 | 21 | 33 | 98 |
|  | -2 (n=5,322), 0 | 158 | 131 | 27 | 95 | 63 | 27 | 36 | 95 |
|  | -1 (n=5,322), 0 | 184 | 134 | 50 | 98 | 86 | 50 | 36 | 98 |
|  | 0 (n=5,322), <8 | 338 | 129 | 209 | 94 | 243 | 209 | 35 | 94 |
|  | 1 (n=5,322), 11 | 286 | 128 | 158 | 93 | 192 | 158 | 35 | 93 |
|  | 2 (n=5,322), 24 | 247 | 125 | 122 | 95 | 152 | 122 | 30 | 95 |
|  | 5 (n=5,322), 31 | 195 | 111 | 84 | 89 | 106 | 84 | 22 | 89 |
|  | 8 (n=5,322), 27 | 178 | 116 | 62 | 92 | 86 | 62 | 24 | 92 |
| 2009 | -8 (n=6637) | 113 | 108 | 4 | 84 | 28 | 4 | 24 | 84 |
|  | -5 (n=6637), <8 | 116 | 107 | 8 | 84 | 32 | 8 | 23 | 84 |
|  | -2 (n=6637), <8 | 133 | 111 | 23 | 87 | 46 | 23 | 24 | 87 |
|  | -1 (n=6637), 8 | 166 | 121 | 44 | 93 | 73 | 44 | 28 | 93 |
|  | 0 (n=6637), 20 | 335 | 134 | 201 | 107 | 228 | 201 | 27 | 107 |
|  | 1 (n=6637), 45 | 268 | 124 | 144 | 99 | 169 | 144 | 25 | 99 |
|  | 2 (n=6637), 91 | 232 | 114 | 118 | 94 | 138 | 118 | 20 | 94 |
|  | 5 (n=6637), 66 | 176 | 98 | 78 | 82 | 94 | 78 | 16 | 82 |
| Cost Category | Home Care | | | | | | | | |
| 2002 | -5 (n=5,322), 0 | 0 | 0 | 0 | 0 | 0 | 0 | 0 | 0 |
|  | -2 (n=5,322), 0 | 0 | 0 | 0 | 0 | 0 | 0 | 0 | 0 |
|  | -1 (n=5,322), 0 | 0 | 0 | 0 | 0 | 0 | 0 | 0 | 0 |
|  | 0 (n=5,322), <8 | 368 | 196 | 172 | 126 | 242 | 172 | 70 | 126 |
|  | 1 (n=5,322), 11 | 454 | 293 | 161 | 208 | 247 | 161 | 85 | 208 |
|  | 2 (n=5,322), 24 | 370 | 271 | 99 | 211 | 159 | 99 | 60 | 211 |
|  | 5 (n=5,322), 31 | 542 | 380 | 162 | 237 | 305 | 162 | 143 | 237 |
|  | 8 (n=5,322), 27 | 556 | 350 | 207 | 280 | 277 | 207 | 70 | 280 |
| 2009 | -8 (n=6637) | 0 | 0 | 0 | 0 | 0 | 0 | 0 | 0 |
|  | -5 (n=6637), <8 | 90 | 103 | -13 | 69 | 21 | -13 | 34 | 69 |
|  | -2 (n=6637), <8 | 204 | 199 | 5 | 117 | 87 | 5 | 82 | 117 |
|  | -1 (n=6637), 8 | 261 | 215 | 46 | 134 | 127 | 46 | 81 | 134 |
|  | 0 (n=6637), 20 | 473 | 286 | 187 | 182 | 292 | 187 | 104 | 182 |
|  | 1 (n=6637), 45 | 548 | 314 | 235 | 210 | 339 | 235 | 104 | 210 |
|  | 2 (n=6637), 91 | 508 | 298 | 210 | 216 | 293 | 210 | 82 | 216 |
|  | 5 (n=6637), 66 | 516 | 331 | 185 | 290 | 226 | 185 | 41 | 290 |
| Cost Category | Rehabilitation | | | | | | | | |
| 2002 | -5 (n=5,322), 0 | 0 | 0 | 0 | 0 | 0 | 0 | 0 | 0 |
|  | -2 (n=5,322), 0 | 0 | 0 | 0 | 0 | 0 | 0 | 0 | 0 |
|  | -1 (n=5,322), 0 | 0 | 0 | 0 | 0 | 0 | 0 | 0 | 0 |
|  | 0 (n=5,322), <8 | 354 | 60 | 293 | 46 | 308 | 293 | 14 | 46 |
|  | 1 (n=5,322), 11 | 226 | 101 | 125 | 115 | 111 | 125 | -14 | 115 |
|  | 2 (n=5,322), 24 | 270 | 109 | 161 | 75 | 195 | 161 | 34 | 75 |
|  | 5 (n=5,322), 31 | 226 | 76 | 149 | 63 | 162 | 149 | 13 | 63 |
|  | 8 (n=5,322), 27 | 175 | 107 | 69 | 92 | 83 | 69 | 15 | 92 |
| 2009 | -8 (n=6637) | 0 | 0 | 0 | 0 | 0 | 0 | 0 | 0 |
|  | -5 (n=6637), <8 | 64 | 29 | 35 | 40 | 24 | 35 | -11 | 40 |
|  | -2 (n=6637), <8 | 89 | 59 | 31 | 39 | 50 | 31 | 20 | 39 |
|  | -1 (n=6637), 8 | 106 | 67 | 39 | 56 | 50 | 39 | 11 | 56 |
|  | 0 (n=6637), 20 | 321 | 73 | 248 | 81 | 240 | 248 | -8 | 81 |
|  | 1 (n=6637), 45 | 248 | 93 | 155 | 79 | 169 | 155 | 14 | 79 |
|  | 2 (n=6637), 91 | 188 | 90 | 98 | 57 | 131 | 98 | 33 | 57 |
|  | 5 (n=6637), 66 | 203 | 88 | 115 | 93 | 110 | 115 | -5 | 93 |
| Cost Category | Emergency Department | | | | | | | | |
| 2002 | -5 (n=5,322), 0 | 0 | 0 | 0 | 0 | 0 | 0 | 0 | 0 |
|  | -2 (n=5,322), 0 | 0 | 0 | 0 | 0 | 0 | 0 | 0 | 0 |
|  | -1 (n=5,322), 0 | 0 | 0 | 0 | 0 | 0 | 0 | 0 | 0 |
|  | 0 (n=5,322), <8 | 0 | 0 | 0 | 0 | 0 | 0 | 0 | 0 |
|  | 1 (n=5,322), 11 | 157 | 114 | 42 | 75 | 81 | 42 | 39 | 75 |
|  | 2 (n=5,322), 24 | 202 | 160 | 42 | 109 | 93 | 42 | 51 | 109 |
|  | 5 (n=5,322), 31 | 207 | 162 | 45 | 105 | 103 | 45 | 57 | 105 |
|  | 8 (n=5,322), 27 | 205 | 159 | 45 | 115 | 89 | 45 | 44 | 115 |
| 2009 | -8 (n=6637) | 0 | 0 | 0 | 0 | 0 | 0 | 0 | 0 |
|  | -5 (n=6637), <8 | 128 | 110 | 18 | 74 | 54 | 18 | 36 | 74 |
|  | -2 (n=6637), <8 | 161 | 130 | 31 | 86 | 76 | 31 | 44 | 86 |
|  | -1 (n=6637), 8 | 187 | 130 | 57 | 91 | 97 | 57 | 39 | 91 |
|  | 0 (n=6637), 20 | 311 | 152 | 159 | 110 | 201 | 159 | 42 | 110 |
|  | 1 (n=6637), 45 | 237 | 156 | 81 | 117 | 120 | 81 | 39 | 117 |
|  | 2 (n=6637), 91 | 227 | 160 | 67 | 116 | 111 | 67 | 44 | 116 |
|  | 5 (n=6637), 66 | 229 | 168 | 61 | 130 | 99 | 61 | 38 | 130 |
| Cost Category | Same Day Surgery | | | | | | | | |
| 2002 | -5 (n=5,322), 0 | 0 | 0 | 0 | 0 | 0 | 0 | 0 | 0 |
|  | -2 (n=5,322), 0 | 0 | 0 | 0 | 0 | 0 | 0 | 0 | 0 |
|  | -1 (n=5,322), 0 | 0 | 0 | 0 | 0 | 0 | 0 | 0 | 0 |
|  | 0 (n=5,322), <8 | 0 | 0 | 0 | 0 | 0 | 0 | 0 | 0 |
|  | 1 (n=5,322), 11 | 123 | 103 | 20 | 75 | 49 | 20 | 28 | 75 |
|  | 2 (n=5,322), 24 | 194 | 147 | 47 | 115 | 78 | 47 | 32 | 115 |
|  | 5 (n=5,322), 31 | 184 | 158 | 26 | 102 | 82 | 26 | 56 | 102 |
|  | 8 (n=5,322), 27 | 179 | 154 | 25 | 109 | 70 | 25 | 45 | 109 |
| 2009 | -8 (n=6637) | 0 | 0 | 0 | 0 | 0 | 0 | 0 | 0 |
|  | -5 (n=6637), <8 | 134 | 139 | -4 | 88 | 46 | -4 | 51 | 88 |
|  | -2 (n=6637), <8 | 187 | 143 | 43 | 114 | 72 | 43 | 29 | 114 |
|  | -1 (n=6637), 8 | 221 | 183 | 38 | 116 | 106 | 38 | 67 | 116 |
|  | 0 (n=6637), 20 | 234 | 166 | 68 | 129 | 105 | 68 | 37 | 129 |
|  | 1 (n=6637), 45 | 215 | 151 | 65 | 130 | 85 | 65 | 21 | 130 |
|  | 2 (n=6637), 91 | 209 | 166 | 43 | 127 | 82 | 43 | 39 | 127 |
|  | 5 (n=6637), 66 | 193 | 164 | 28 | 127 | 65 | 28 | 37 | 127 |
| Cost Category | Continuing Care | | | | | | | | |
| 2002 | -5 (n=5,322), 0 | 0 | 0 | 0 | 0 | 0 | 0 | 0 | 0 |
|  | -2 (n=5,322), 0 | 0 | 0 | 0 | 0 | 0 | 0 | 0 | 0 |
|  | -1 (n=5,322), 0 | 0 | 0 | 0 | 0 | 0 | 0 | 0 | 0 |
|  | 0 (n=5,322), <8 | 54 | 92 | -39 | 9 | 45 | -39 | 83 | 9 |
|  | 1 (n=5,322), 11 | 279 | 137 | 143 | 136 | 143 | 143 | 1 | 136 |
|  | 2 (n=5,322), 24 | 299 | 107 | 192 | 181 | 118 | 192 | -74 | 181 |
|  | 5 (n=5,322), 31 | 137 | 226 | -89 | 126 | 11 | -89 | 100 | 126 |
|  | 8 (n=5,322), 27 | 135 | 357 | -222 | 108 | 27 | -222 | 249 | 108 |
| 2009 | -8 (n=6637) | 0 | 0 | 0 | 0 | 0 | 0 | 0 | 0 |
|  | -5 (n=6637), <8 | 3 | 59 | -57 | 35 | -32 | -57 | 24 | 35 |
|  | -2 (n=6637), <8 | 35 | 84 | -49 | 6 | 29 | -49 | 78 | 6 |
|  | -1 (n=6637), 8 | 44 | 78 | -34 | 33 | 11 | -34 | 45 | 33 |
|  | 0 (n=6637), 20 | 100 | 55 | 44 | 49 | 50 | 44 | 6 | 49 |
|  | 1 (n=6637), 45 | 201 | 77 | 124 | 111 | 90 | 124 | -34 | 111 |
|  | 2 (n=6637), 91 | 178 | 162 | 16 | 90 | 88 | 16 | 72 | 90 |
|  | 5 (n=6637), 66 | 108 | 162 | -54 | 144 | -36 | -54 | 18 | 144 |
| Cost Category | Long-Term Care | | | | | | | | |
| 2002 | -5 (n=5,322), 0 | 0 | 0 | 0 | 0 | 0 | 0 | 0 | 0 |
|  | -2 (n=5,322), 0 | 0 | 0 | 0 | 0 | 0 | 0 | 0 | 0 |
|  | -1 (n=5,322), 0 | 0 | 0 | 0 | 0 | 0 | 0 | 0 | 0 |
|  | 0 (n=5,322), <8 | 165 | 326 | -160 | 262 | -97 | -160 | 64 | 262 |
|  | 1 (n=5,322), 11 | 424 | 522 | -98 | 393 | 32 | -98 | 129 | 393 |
|  | 2 (n=5,322), 24 | 509 | 529 | -21 | 433 | 76 | -21 | 96 | 433 |
|  | 5 (n=5,322), 31 | 668 | 602 | 65 | 674 | -6 | 65 | -72 | 674 |
|  | 8 (n=5,322), 27 | 612 | 667 | -54 | 691 | -79 | -54 | -24 | 691 |
| 2009 | -8 (n=6637) | 0 | 0 | 0 | 0 | 0 | 0 | 0 | 0 |
|  | -5 (n=6637), <8 | 15 | 80 | -65 | 41 | -26 | -65 | 39 | 41 |
|  | -2 (n=6637), <8 | 37 | 192 | -155 | 161 | -124 | -155 | 31 | 161 |
|  | -1 (n=6637), 8 | 53 | 225 | -172 | 232 | -179 | -172 | -7 | 232 |
|  | 0 (n=6637), 20 | 113 | 295 | -181 | 316 | -203 | -181 | -21 | 316 |
|  | 1 (n=6637), 45 | 264 | 421 | -157 | 375 | -111 | -157 | 46 | 375 |
|  | 2 (n=6637), 91 | 367 | 496 | -130 | 380 | -14 | -130 | 116 | 380 |
|  | 5 (n=6637), 66 | 478 | 549 | -70 | 452 | 26 | -70 | 97 | 452 |
| Cost Category | Capitation | | | | | | | | |
| 2002 | -5 (n=5,322), 0 | 0 | 0 | 0 | 0 | 0 | 0 | 0 | 0 |
|  | -2 (n=5,322), 0 | 0 | 0 | 0 | 0 | 0 | 0 | 0 | 0 |
|  | -1 (n=5,322), 0 | 0 | 0 | 0 | 0 | 0 | 0 | 0 | 0 |
|  | 0 (n=5,322), <8 | 0 | 0 | 0 | 0 | 0 | 0 | 0 | 0 |
|  | 1 (n=5,322), 11 | 0 | 0 | 0 | 0 | 0 | 0 | 0 | 0 |
|  | 2 (n=5,322), 24 | 0 | 0 | 0 | 0 | 0 | 0 | 0 | 0 |
|  | 5 (n=5,322), 31 | 64 | 65 | -1 | 56 | 8 | -1 | 9 | 56 |
|  | 8 (n=5,322), 27 | 123 | 123 | 0 | 114 | 9 | 0 | 9 | 114 |
| 2009 | -8 (n=6637) | 0 | 0 | 0 | 0 | 0 | 0 | 0 | 0 |
|  | -5 (n=6637), <8 | 0 | 0 | 0 | 0 | 0 | 0 | 0 | 0 |
|  | -2 (n=6637), <8 | 64 | 60 | 4 | 52 | 12 | 4 | 8 | 52 |
|  | -1 (n=6637), 8 | 77 | 71 | 6 | 63 | 14 | 6 | 8 | 63 |
|  | 0 (n=6637), 20 | 121 | 109 | 12 | 97 | 24 | 12 | 12 | 97 |
|  | 1 (n=6637), 45 | 143 | 128 | 15 | 113 | 30 | 15 | 15 | 113 |
|  | 2 (n=6637), 91 | 159 | 142 | 17 | 126 | 33 | 17 | 16 | 126 |
|  | 5 (n=6637), 66 | 169 | 152 | 17 | 138 | 31 | 17 | 14 | 138 |
| Cost Category | Cancer Clinics | | | | | | | | |
| 2002 | -5 (n=5,322), 0 | 0 | 0 | 0 | 0 | 0 | 0 | 0 | 0 |
|  | -2 (n=5,322), 0 | 0 | 0 | 0 | 0 | 0 | 0 | 0 | 0 |
|  | -1 (n=5,322), 0 | 0 | 0 | 0 | 0 | 0 | 0 | 0 | 0 |
|  | 0 (n=5,322), <8 | 0 | 0 | 0 | 0 | 0 | 0 | 0 | 0 |
|  | 1 (n=5,322), 11 | 0 | 0 | 0 | 0 | 0 | 0 | 0 | 0 |
|  | 2 (n=5,322), 24 | 0 | 0 | 0 | 0 | 0 | 0 | 0 | 0 |
|  | 5 (n=5,322), 31 | 122 | 78 | 44 | 97 | 25 | 44 | -19 | 97 |
|  | 8 (n=5,322), 27 | 73 | 92 | -19 | 75 | -2 | -19 | 17 | 75 |
| 2009 | -8 (n=6637) | 0 | 0 | 0 | 0 | 0 | 0 | 0 | 0 |
|  | -5 (n=6637), <8 | 0 | 0 | 0 | 0 | 0 | 0 | 0 | 0 |
|  | -2 (n=6637), <8 | 62 | 67 | -5 | 61 | 1 | -5 | 6 | 61 |
|  | -1 (n=6637), 8 | 64 | 53 | 11 | 61 | 2 | 11 | -8 | 61 |
|  | 0 (n=6637), 20 | 83 | 80 | 3 | 62 | 21 | 3 | 18 | 62 |
|  | 1 (n=6637), 45 | 106 | 95 | 11 | 87 | 19 | 11 | 8 | 87 |
|  | 2 (n=6637), 91 | 193 | 162 | 31 | 115 | 78 | 31 | 47 | 115 |
|  | 5 (n=6637), 66 | 129 | 157 | -29 | 128 | 0 | -29 | 29 | 128 |
| Cost Category | Dialysis Clinics | | | | | | | | |
| 2002 | -5 (n=5,322), 0 | 0 | 0 | 0 | 0 | 0 | 0 | 0 | 0 |
|  | -2 (n=5,322), 0 | 0 | 0 | 0 | 0 | 0 | 0 | 0 | 0 |
|  | -1 (n=5,322), 0 | 0 | 0 | 0 | 0 | 0 | 0 | 0 | 0 |
|  | 0 (n=5,322), <8 | 0 | 0 | 0 | 0 | 0 | 0 | 0 | 0 |
|  | 1 (n=5,322), 11 | 0 | 0 | 0 | 0 | 0 | 0 | 0 | 0 |
|  | 2 (n=5,322), 24 | 0 | 0 | 0 | 0 | 0 | 0 | 0 | 0 |
|  | 5 (n=5,322), 31 | 195 | 126 | 69 | 11 | 184 | 69 | 115 | 11 |
|  | 8 (n=5,322), 27 | 122 | 103 | 19 | 48 | 74 | 19 | 55 | 48 |
| 2009 | -8 (n=6637) | 0 | 0 | 0 | 0 | 0 | 0 | 0 | 0 |
|  | -5 (n=6637), <8 | 0 | 0 | 0 | 0 | 0 | 0 | 0 | 0 |
|  | -2 (n=6637), <8 | 71 | 70 | 1 | 39 | 32 | 1 | 31 | 39 |
|  | -1 (n=6637), 8 | 99 | 94 | 5 | 58 | 42 | 5 | 36 | 58 |
|  | 0 (n=6637), 20 | 120 | 145 | -25 | 76 | 44 | -25 | 69 | 76 |
|  | 1 (n=6637), 45 | 153 | 161 | -8 | 93 | 61 | -8 | 68 | 93 |
|  | 2 (n=6637), 91 | 117 | 90 | 27 | 86 | 31 | 27 | 4 | 86 |
|  | 5 (n=6637), 66 | 117 | 135 | -18 | 64 | 53 | -18 | 71 | 64 |
| Cost Category | Mental Health Inpatient | | | | | | | | |
| 2002 | -5 (n=5,322), 0 | 0 | 0 | 0 | 0 | 0 | 0 | 0 | 0 |
|  | -2 (n=5,322), 0 | 0 | 0 | 0 | 0 | 0 | 0 | 0 | 0 |
|  | -1 (n=5,322), 0 | 0 | 0 | 0 | 0 | 0 | 0 | 0 | 0 |
|  | 0 (n=5,322), <8 | 0 | 0 | 0 | 0 | 0 | 0 | 0 | 0 |
|  | 1 (n=5,322), 11 | 0 | 0 | 0 | 0 | 0 | 0 | 0 | 0 |
|  | 2 (n=5,322), 24 | 0 | 0 | 0 | 0 | 0 | 0 | 0 | 0 |
|  | 5 (n=5,322), 31 | 59 | 137 | -77 | 23 | 37 | -77 | 114 | 23 |
|  | 8 (n=5,322), 27 | 40 | 168 | -128 | 49 | -9 | -128 | 119 | 49 |
| 2009 | -8 (n=6637) | 0 | 0 | 0 | 0 | 0 | 0 | 0 | 0 |
|  | -5 (n=6637), <8 | 0 | 0 | 0 | 0 | 0 | 0 | 0 | 0 |
|  | -2 (n=6637), <8 | 64 | 88 | -23 | 41 | 24 | -23 | 47 | 41 |
|  | -1 (n=6637), 8 | 45 | 71 | -26 | 70 | -25 | -26 | 1 | 70 |
|  | 0 (n=6637), 20 | 50 | 48 | 2 | 91 | -41 | 2 | -43 | 91 |
|  | 1 (n=6637), 45 | 61 | 53 | 8 | 102 | -40 | 8 | -49 | 102 |
|  | 2 (n=6637), 91 | 73 | 41 | 33 | 162 | -89 | 33 | -121 | 162 |
|  | 5 (n=6637), 66 | 89 | 29 | 60 | 149 | -59 | 60 | -120 | 149 |
| Cost Category | Assistive Devices | | | | | | | | |
| 2002 | -5 (n=5,322), 0 | 0 | 0 | 0 | 0 | 0 | 0 | 0 | 0 |
|  | -2 (n=5,322), 0 | 0 | 0 | 0 | 0 | 0 | 0 | 0 | 0 |
|  | -1 (n=5,322), 0 | 0 | 0 | 0 | 0 | 0 | 0 | 0 | 0 |
|  | 0 (n=5,322), <8 | 0 | 0 | 0 | 0 | 0 | 0 | 0 | 0 |
|  | 1 (n=5,322), 11 | 0 | 0 | 0 | 0 | 0 | 0 | 0 | 0 |
|  | 2 (n=5,322), 24 | 34 | 15 | 19 | 13 | 21 | 19 | 2 | 13 |
|  | 5 (n=5,322), 31 | 54 | 33 | 21 | 26 | 28 | 21 | 7 | 26 |
|  | 8 (n=5,322), 27 | 9 | 10 | 0 | 6 | 3 | 0 | 4 | 6 |
| 2009 | -8 (n=6637) | 0 | 0 | 0 | 0 | 0 | 0 | 0 | 0 |
|  | -5 (n=6637), <8 | 7 | 8 | -2 | 8 | -1 | -2 | 0 | 8 |
|  | -2 (n=6637), <8 | 18 | 22 | -4 | 12 | 6 | -4 | 10 | 12 |
|  | -1 (n=6637), 8 | 25 | 18 | 7 | 19 | 6 | 7 | -1 | 19 |
|  | 0 (n=6637), 20 | 36 | 21 | 16 | 20 | 16 | 16 | 1 | 20 |
|  | 1 (n=6637), 45 | 12 | 8 | 4 | 6 | 6 | 4 | 2 | 6 |
|  | 2 (n=6637), 91 | 0 | 0 | 0 | 0 | 0 | 0 | 0 | 0 |
|  | 5 (n=6637), 66 | 0 | 0 | 0 | 0 | 0 | 0 | 0 | 0 |
