# Supplementary material for: Health care costs of rheumatoid arthritis: A longitudinal population study
Source: PLoS One. 2021 May 6;16(5):e0251334. doi: 10.1371/journal.pone.0251334 (PMC8101709; doi:10.1371/journal.pone.0251334)
Supplement: S3 Table — (DOCX) [file pone.0251334.s004.docx]

Supplement 5: Demographics and Costs by Exposure Group and Year ^a^

| Years Before and After Diagnosis | -8 | -8 | -8 | -5 | -5 | -5 | -2 | -2 | -2 | -1 | -1 | -1 | 0 | 0 | 0 | 1 | 1 | 1 | 2 | 2 | 2 | 5 | 5 | 5 | 8 | 8 | 8 |
| --- | --- | --- | --- | --- | --- | --- | --- | --- | --- | --- | --- | --- | --- | --- | --- | --- | --- | --- | --- | --- | --- | --- | --- | --- | --- | --- | --- |
| Exposure Group | Case^b^ | Control^c^ | Random Control^d^ | Case | Control | Random Control | Case | Control | Random Control | Case | Control | Random Control | Case | Control | Random Control | Case | Control | Random Control | Case | Control | Random Control | Case | Control | Random Control | Case | Control | Random Control |
| Age  Mean, Median, SD, (range) | - | - | - | - | - | - | - | - | - | - | - | - | 56.8,58,16.7, (15-99) | 56.8,58,16.7, (15-99) | 56.8,58,16.7, (15-99) | - | - | - | - | - | - | - | - | - | - | - | - |
| Female % | - | - | - | - | - | - | - | - | - | - | - | - | 70 | 70 | 70 | - | - | - | - | - | - | - | - | - | - | - | - |
| Incident Year  Mean, Median, SD, (range) | - | - | - | - | - | - | - | - | - | - | - | - | 2006,2007,6.3.1995-2016 | - | - | - | - | - | - | - | - | - | - | - | - | - | - |
| Number of Patients | 94191 | 93282 | 93707 | 104933 | 103853 | 104332 | 104933 | 103853 | 104332 | 104933 | 103853 | 104332 | 104933 | 103853 | 104333 | 98751 | 97900 | 98214 | 91119 | 90418 | 90656 | 67650 | 67237 | 67393 | 46901 | 46691 | 46788 |
| Number of Patients on Biologics | 8 |  |  | 34 |  |  | 94 |  |  | 137 |  |  | 506 |  |  | 1007 |  |  | 1037 |  |  | 577 |  |  | 412 |  |  |
|  |  |  |  |  |  |  |  |  |  |  |  |  |  |  |  |  |  |  |  |  |  |  |  |  |  |  |  |
| Inpatient Care  Total in millions, (per patient), % of total cost, %∆in cost | 27.8,295.1,%16.4,NA | 24.8,266.4,%15.1,%Δ11 | 17.1,182,%14.8,%Δ62 | 43.6,415.7,%17.3,NA | 43,414.1,%17.2,%Δ0 | 28.7,275,%16.6,%Δ51 | 77.3,736.2,%20.5,NA | 70.9,682.6,%20.4,%Δ8 | 48.3,463,%19.3,%Δ59 | 101.1,963.2,%22,NA | 82.6,795.4,%20.8,%Δ21 | 56.5,541.5,%19.9,%Δ78 | 280.5,2673.2,%31.6,NA | 97.2,936.4,%21.2,%Δ185 | 68.4,655.3,%20.7,%Δ308 | 213.8,2164.9,%26.9,NA | 116.6,1191.3,%23.6,%Δ82 | 87.7,893.2,%23.7,%Δ142 | 178.3,1957.3,%24.9,NA | 107.6,1190.1,%23.1,%Δ64 | 83.1,916.2,%23.4,%Δ114 | 125.8,1860.1,%23.3,NA | 78.9,1173.1,%21.7,%Δ56 | 66.5,986.2,%23.1,%Δ103 | 87.2,1858.4,%23.1,NA | 58.7,1256.2,%22.8,%Δ58 | 46.7,998.6,%22.9,%Δ88 |
| Physician Fee for Service Billings (Specialist) | 53.6,569.1,%31.7,NA | 50.5,541.2,%30.7,%Δ5 | 34.7,370.5,%30.1,%Δ54 | 68.7,655.2,%27.3,NA | 63.4,610.8,%25.4,%Δ7 | 43.7,418.5,%25.2,%Δ57 | 84.3,803.5,%22.4,NA | 69,664.5,%19.8,%Δ21 | 49.4,473.9,%19.8,%Δ70 | 97.3,927.3,%21.2,NA | 71.3,686.8,%18,%Δ35 | 51.4,492.6,%18.1,%Δ88 | 173.9,1656.9,%19.6,NA | 73.9,711.8,%16.1,%Δ133 | 53.5,512.4,%16.2,%Δ223 | 134.4,1360.9,%16.9,NA | 71.8,733.9,%14.6,%Δ85 | 53.2,541.8,%14.4,%Δ151 | 112.5,1234.9,%15.7,NA | 65.3,722.6,%14,%Δ71 | 49.6,547.4,%14,%Δ126 | 74.6,1102.9,%13.8,NA | 47.9,712.2,%13.2,%Δ53 | 37.5,557.1,%13.1,%Δ101 | 48.3,1030.2,%12.8,NA | 32.9,705.7,%12.8,%Δ45 | 25.8,550.6,%12.6,%Δ85 |
| Drug Benefits | 24.2,256.6,%14.3,NA | 24.2,259.5,%14.7,%Δ-1 | 17.2,183.5,%14.9,%Δ40 | 40.1,382.6,%15.9,NA | 38.3,369.3,%15.3,%Δ4 | 27.4,262.3,%15.8,%Δ46 | 61.9,589.5,%16.4,NA | 54.1,520.9,%15.5,%Δ13 | 39.8,381.2,%15.9,%Δ55 | 73.1,696.8,%15.9,NA | 60.4,581.7,%15.2,%Δ20 | 44.3,424.3,%15.6,%Δ64 | 100.6,958.5,%11.3,NA | 67.3,648.5,%14.7,%Δ48 | 49.9,478.2,%15.1,%Δ100 | 122.7,1243,%15.4,NA | 67.9,693.5,%13.8,%Δ79 | 51.4,523.6,%13.9,%Δ137 | 129,1416.1,%18,NA | 65.8,727.3,%14.1,%Δ95 | 49.4,545.4,%14,%Δ160 | 112.2,1658.6,%20.8,NA | 49.7,739.7,%13.7,%Δ128 | 39.2,581,%13.6,%Δ204 | 82.2,1751.8,%21.8,NA | 34.8,744.7,%13.5,%Δ137 | 28.7,613.5,%14.1,%Δ202 |
| Outpatient | 6.4,67.7,%3.8,NA | 5.8,61.7,%3.5,%Δ10 | 4.1,43.4,%3.5,%Δ56 | 15.4,146.5,%6.1,NA | 12.9,123.8,%5.1,%Δ18 | 9.4,90.5,%5.5,%Δ62 | 28.2,269,%7.5,NA | 20.5,197.8,%5.9,%Δ36 | 14.7,141.2,%5.9,%Δ91 | 35.1,334.1,%7.6,NA | 22.8,219.7,%5.7,%Δ52 | 16.9,162.1,%6,%Δ106 | 64.9,618.4,%7.3,NA | 26.2,252.2,%5.7,%Δ145 | 19.3,184.8,%5.8,%Δ235 | 59.1,598.7,%7.4,NA | 27.6,281.5,%5.6,%Δ113 | 20.3,206.8,%5.5,%Δ189 | 51.4,564.5,%7.2,NA | 26.7,295.4,%5.7,%Δ91 | 19.8,218.6,%5.6,%Δ158 | 39.4,581.8,%7.3,NA | 23.4,348.3,%6.5,%Δ97 | 18.1,268.5,%6.3,%Δ166 | 24.4,519.4,%6.5,NA | 15.5,331.3,%6,%Δ49 | 12.2,260.5,%6,%Δ93 |
| Physician Fee for Service Billings (General Practitioner) | 26.3,279.2,%15.5,NA | 27.2,291.2,%16.5,%Δ-4 | 19,202.3,%16.4,%Δ38 | 29.5,281.5,%11.7,NA | 30.2,291.3,%12.1,%Δ-3 | 21,201.7,%12.2,%Δ40 | 30,285.9,%8,NA | 28.5,274.6,%8.2,%Δ4 | 21,201.3,%8.4,%Δ42 | 32.1,305.5,%7,NA | 28.9,278.1,%7.3,%Δ10 | 20.7,198,%7.3,%Δ54 | 43.5,414.1,%4.9,NA | 28.7,276.2,%6.2,%Δ50 | 20.4,195.5,%6.2,%Δ112 | 34.8,352.3,%4.4,NA | 26.9,275.2,%5.5,%Δ28 | 19.8,201.5,%5.3,%Δ75 | 29.4,322.4,%4.1,NA | 24.3,268.7,%5.2,%Δ20 | 17.8,196.7,%5,%Δ64 | 19.3,286,%3.6,NA | 17,252.2,%4.7,%Δ6 | 12.7,188.4,%4.4,%Δ45 | 12,255.1,%3.2,NA | 10.4,222.8,%4,%Δ1 | 8.2,174.3,%4,%Δ35 |
| Laboratory | 3.7,39.4,%2.2,NA | 4.1,44.3,%2.5,%Δ-11 | 2.7,28.8,%2.3,%Δ37 | 8.1,76.7,%3.2,NA | 8.4,80.6,%3.3,%Δ-5 | 5.5,52.8,%3.2,%Δ45 | 16.5,157.5,%4.4,NA | 15.9,153,%4.6,%Δ3 | 10.9,104.8,%4.4,%Δ50 | 23.2,221.1,%5,NA | 19.8,190.9,%5,%Δ16 | 13.6,130.5,%4.8,%Δ69 | 43.1,410.9,%4.9,NA | 24.9,240.1,%5.4,%Δ71 | 17.7,169.8,%5.4,%Δ142 | 46.8,474.1,%5.9,NA | 28.4,290.6,%5.8,%Δ63 | 20.9,212.4,%5.6,%Δ123 | 43.8,480.7,%6.1,NA | 27.7,305.9,%5.9,%Δ57 | 20.6,226.8,%5.8,%Δ112 | 34,502.5,%6.3,NA | 21.9,326.1,%6,%Δ64 | 17.1,254,%6,%Δ122 | 23.8,507.2,%6.3,NA | 16.6,354.7,%6.4,%Δ56 | 13.5,287.8,%6.6,%Δ100 |
| Home Care Services | 10.6,112.7,%6.3,NA | 10.1,108.1,%6.1,%Δ4 | 7.5,79.7,%6.5,%Δ41 | 13.1,125,%5.2,NA | 12,115.3,%4.8,%Δ8 | 8.9,85,%5.1,%Δ47 | 14.8,141.3,%3.9,NA | 12.1,116.5,%3.5,%Δ21 | 9.1,87.3,%3.6,%Δ62 | 17.1,163.4,%3.7,NA | 12.2,117.7,%3.1,%Δ39 | 9.2,88,%3.2,%Δ86 | 32.3,307.7,%3.6,NA | 12.4,119.3,%2.7,%Δ158 | 9.4,89.9,%2.8,%Δ242 | 25.3,256.6,%3.2,NA | 11.6,118.2,%2.3,%Δ117 | 8.9,90.6,%2.4,%Δ183 | 20.8,228.2,%2.9,NA | 10.5,116,%2.2,%Δ97 | 8.2,90.6,%2.3,%Δ152 | 12.8,188.9,%2.4,NA | 7.5,111,%2.1,%Δ63 | 6.1,90,%2.1,%Δ109 | 7.8,166.8,%2.1,NA | 4.9,104.5,%1.9,%Δ50 | 4,86.4,%2,%Δ85 |
| Rehabilitation | 2.1,22.3,%1.2,NA | 1.7,18.3,%1,%Δ22 | 1.5,16.4,%1.3,%Δ36 | 3.5,32.9,%1.4,NA | 3.5,34.1,%1.4,%Δ-3 | 2.3,22.3,%1.3,%Δ48 | 6.4,60.7,%1.7,NA | 5.8,56.3,%1.7,%Δ8 | 4.3,40.8,%1.7,%Δ49 | 8.7,82.8,%1.9,NA | 6.6,64,%1.7,%Δ29 | 5.4,51.7,%1.9,%Δ60 | 31.2,297.4,%3.5,NA | 8.5,82.3,%1.9,%Δ261 | 6.2,59.8,%1.9,%Δ397 | 21.2,214.8,%2.7,NA | 8.5,86.8,%1.7,%Δ147 | 7.5,76.5,%2,%Δ181 | 17.3,190.3,%2.4,NA | 9,99.1,%1.9,%Δ92 | 7,77.7,%2,%Δ145 | 12.8,189.4,%2.4,NA | 6.4,94.9,%1.8,%Δ91 | 5.6,83.4,%2,%Δ144 | 7.7,164.2,%2,NA | 4.5,95.9,%1.7,%Δ73 | 4.2,89.2,%2,%Δ97 |
| Emergency Department | 4.7,49.5,%2.8,NA | 4.4,47.3,%2.7,%Δ5 | 2.8,30.3,%2.5,%Δ64 | 8.1,77.1,%3.2,NA | 7.3,69.9,%2.9,%Δ10 | 4.8,46.2,%2.8,%Δ67 | 12.9,123,%3.4,NA | 10.8,104.3,%3.1,%Δ18 | 7.3,69.9,%2.9,%Δ76 | 16.5,157.5,%3.6,NA | 12.3,118.8,%3.1,%Δ33 | 8.3,79.3,%2.9,%Δ99 | 28.5,271.4,%3.2,NA | 14.1,135.9,%3.1,%Δ100 | 9.5,91,%2.9,%Δ198 | 21.3,216,%2.7,NA | 14.7,149.7,%3,%Δ44 | 10.5,107.4,%2.8,%Δ101 | 20,219.3,%2.8,NA | 14.5,159.8,%3.1,%Δ37 | 10.4,115.1,%2.9,%Δ91 | 14.2,209.8,%2.6,NA | 10.9,161.9,%3,%Δ31 | 8.1,119.6,%2.8,%Δ82 | 9.5,202.6,%2.5,NA | 7.5,161.3,%2.9,%Δ25 | 5.7,122.6,%2.8,%Δ69 |
| Same Day Surgery | 5.6,59.3,%3.3,NA | 4.8,51.2,%2.9,%Δ16 | 3.8,40.3,%3.3,%Δ47 | 10,95.1,%4,NA | 8.5,82.2,%3.4,%Δ16 | 6.2,59,%3.6,%Δ61 | 15.3,145.9,%4.1,NA | 12.1,116.2,%3.5,%Δ26 | 9.2,87.9,%3.7,%Δ66 | 17.6,167.6,%3.8,NA | 13.6,131.3,%3.4,%Δ28 | 10,95.6,%3.5,%Δ75 | 21.2,202.3,%2.4,NA | 14.8,142.9,%3.2,%Δ42 | 11.2,107.2,%3.4,%Δ89 | 20.2,204.9,%2.5,NA | 14.7,150.4,%3,%Δ36 | 11.4,115.9,%3.1,%Δ77 | 19.1,210,%2.7,NA | 14.4,159,%3.1,%Δ32 | 11.1,122.2,%3.1,%Δ72 | 13.3,197.1,%2.5,NA | 10.6,157.3,%2.9,%Δ24 | 8.2,122.3,%2.9,%Δ61 | 8.5,181.9,%2.3,NA | 7.2,153.3,%2.8,%Δ16 | 5.5,117.7,%2.7,%Δ49 |
| Complex and Continuing Care | 0.2,1.7,%0.1,NA | 0.9,9.9,%0.6,%Δ-83 | 0.8,9,%0.7,%Δ-81 | 0.8,7.8,%0.3,NA | 3.1,30.2,%1.3,%Δ-74 | 1.3,12.6,%0.8,%Δ-38 | 2.8,26.8,%0.7,NA | 6.4,61.5,%1.8,%Δ-56 | 3.8,36.8,%1.5,%Δ-27 | 5.1,48.2,%1.1,NA | 10.3,98.8,%2.6,%Δ-51 | 5.6,54,%2,%Δ-11 | 17.9,170.8,%2,NA | 15,144.2,%3.3,%Δ18 | 7.7,74.1,%2.3,%Δ130 | 24,242.7,%3,NA | 16.5,169,%3.4,%Δ44 | 12.4,126.3,%3.4,%Δ92 | 20.2,221.4,%2.8,NA | 15.3,169.3,%3.3,%Δ31 | 12.4,136.3,%3.5,%Δ62 | 13.2,194.6,%2.4,NA | 11.8,175.5,%3.3,%Δ15 | 9.2,136,%3.2,%Δ43 | 11.9,253,%3.1,NA | 9.7,208,%3.8,%Δ44 | 5.8,123.4,%2.8,%Δ86 |
| Long-Term Care | 0.3,3.6,%0.2,NA | 1.7,18.8,%1.1,%Δ-81 | 1.3,14,%1.1,%Δ-74 | 1.4,13.4,%0.6,NA | 6.1,59,%2.4,%Δ-77 | 4.2,40.4,%2.4,%Δ-67 | 4.5,42.8,%1.2,NA | 17.9,172.2,%5.1,%Δ-75 | 15.1,144.9,%6,%Δ-70 | 6.7,64,%1.5,NA | 27,259.7,%6.8,%Δ-75 | 21.5,206.5,%7.6,%Δ-69 | 16.4,156.7,%1.9,NA | 38.8,374,%8.5,%Δ-58 | 31,296.8,%9.4,%Δ-47 | 33.6,339.9,%4.2,NA | 49.3,503.2,%10,%Δ-32 | 38.2,388.8,%10.3,%Δ-13 | 37.3,409,%5.2,NA | 49.1,542.8,%10.5,%Δ-25 | 36.9,406.8,%10.4,%Δ1 | 35.4,523.8,%6.6,NA | 42.6,633.2,%11.7,%Δ-4 | 33.6,499.3,%11.7,%Δ29 | 29,618.1,%7.7,NA | 30.3,649.7,%11.8,%Δ-2 | 26.1,558.7,%12.8,%Δ24 |
| Capitation Costs (Family Health Teams) | 1.3,13.5,%0.7,NA | 1,11.1,%0.6,%Δ21 | 1,10.6,%0.9,%Δ27 | 4.1,39.3,%1.6,NA | 3.5,33.7,%1.4,%Δ17 | 3.3,31.3,%1.9,%Δ26 | 7.8,74.2,%2.1,NA | 6.8,65.4,%2,%Δ13 | 6.2,59.6,%2.5,%Δ25 | 9.1,86.8,%2,NA | 8,76.8,%2,%Δ13 | 7.3,69.6,%2.6,%Δ25 | 10.5,100.1,%1.2,NA | 9.1,87.8,%2,%Δ14 | 8.3,79.5,%2.5,%Δ26 | 10.7,108.1,%1.3,NA | 9.3,94.7,%1.9,%Δ14 | 8.4,85.6,%2.3,%Δ26 | 10.3,113.6,%1.4,NA | 9.1,100.3,%1.9,%Δ13 | 8.2,90.8,%2.3,%Δ25 | 8.9,131.1,%1.6,NA | 8.1,120.4,%2.2,%Δ31 | 7.4,109.8,%2.6,%Δ44 | 6.7,143.2,%1.8,NA | 6.4,136.6,%2.5,%Δ19 | 5.9,125.7,%2.9,%Δ30 |
| Cancer Clinics | 0.9,9.7,%0.5,NA | 0.6,6.9,%0.4,%Δ42 | 0.8,8.7,%0.7,%Δ12 | 1.9,17.6,%0.7,NA | 2.1,20.2,%0.8,%Δ-12 | 1.7,16.4,%1,%Δ8 | 4.2,40.2,%1.1,NA | 4.9,46.7,%1.4,%Δ-14 | 4.2,39.8,%1.7,%Δ1 | 6.2,58.7,%1.3,NA | 6,57.4,%1.5,%Δ2 | 5.6,54.1,%2,%Δ9 | 8.7,82.8,%1,NA | 8.4,80.5,%1.8,%Δ3 | 7.9,75.8,%2.4,%Δ9 | 9.5,96.5,%1.2,NA | 9.2,94.4,%1.9,%Δ2 | 8.2,83.3,%2.2,%Δ16 | 10.4,114.2,%1.5,NA | 8.9,98.2,%1.9,%Δ16 | 7.8,86.4,%2.2,%Δ32 | 8.8,130.7,%1.6,NA | 8.2,122.1,%2.3,%Δ33 | 7,103.9,%2.4,%Δ51 | 6.4,136.9,%1.7,NA | 5.4,116.3,%2.1,%Δ12 | 5.3,112.9,%2.6,%Δ32 |
| Dialysis Clinics | 0.5,5,%0.3,NA | 0.8,8.3,%0.5,%Δ-39 | 0.3,3.2,%0.3,%Δ57 | 1.8,16.9,%0.7,NA | 2,19.2,%0.8,%Δ-12 | 1.3,12.9,%0.8,%Δ31 | 4.9,46.4,%1.3,NA | 4.5,42.9,%1.3,%Δ8 | 2.8,26.7,%1.1,%Δ74 | 6,56.9,%1.3,NA | 6.9,66,%1.7,%Δ-14 | 3.5,33.5,%1.2,%Δ70 | 8.7,82.8,%1,NA | 9.6,92.9,%2.1,%Δ-11 | 4.9,47.1,%1.5,%Δ76 | 10.3,104.2,%1.3,NA | 9.8,99.7,%2,%Δ5 | 6.1,61.7,%1.6,%Δ69 | 9.5,104.1,%1.3,NA | 8.9,98.2,%1.9,%Δ6 | 5.7,62.4,%1.6,%Δ67 | 9.6,141.5,%1.8,NA | 9.3,138,%2.6,%Δ44 | 5.8,85.6,%2,%Δ127 | 6.9,146.1,%1.8,NA | 6,128.4,%2.3,%Δ6 | 3.4,72.5,%1.7,%Δ71 |
| Mental Health Inpatient Care | 0.8,8.7,%0.5,NA | 1.5,16.5,%0.9,%Δ-47 | 0.5,5.8,%0.5,%Δ49 | 1.5,14.1,%0.6,NA | 5.1,48.8,%2,%Δ-71 | 3,28.7,%1.7,%Δ-51 | 3.6,34.5,%1,NA | 7.1,68.4,%2,%Δ-50 | 3.4,32.9,%1.4,%Δ5 | 3.7,35.4,%0.8,NA | 7.7,73.9,%1.9,%Δ-52 | 3.6,34.3,%1.3,%Δ3 | 5.2,49.6,%0.6,NA | 9.1,87.4,%2,%Δ-43 | 4.6,44.3,%1.4,%Δ12 | 5.6,56.4,%0.7,NA | 9.6,98,%1.9,%Δ-42 | 4.7,47.7,%1.3,%Δ18 | 5.8,63.7,%0.8,NA | 8.2,90.9,%1.8,%Δ-30 | 5.5,61,%1.6,%Δ4 | 5.3,77.7,%1,NA | 7.8,116.7,%2.2,%Δ-14 | 4.6,67.9,%1.6,%Δ27 | 4.4,94.1,%1.2,NA | 6.2,133,%2.4,%Δ-19 | 3.1,65.4,%1.5,%Δ39 |
| Assistive Devices | 0.4,3.8,%0.2,NA | 0.5,4.8,%0.3,%Δ-22 | 0.3,2.7,%0.2,%Δ40 | 0.7,6.6,%0.3,NA | 0.7,6.7,%0.3,Δ-1 | 0.4,4.1,%0.2,%Δ60 | 0.8,7.7,%0.2,NA | 0.8,8.1,%0.2,%Δ-5 | 0.6,5.4,%0.2,%Δ43 | 1,9.8,%0.2,NA | 0.9,8.9,%0.2,%Δ11 | 0.7,6.9,%0.3,%Δ42 | 1.5,14.7,%0.2,NA | 1.1,10.4,%0.2,%Δ42 | 0.7,6.7,%0.2,%Δ120 | 1.9,19.1,%0.2,NA | 1.1,11.4,%0.2,%Δ67 | 0.7,7.2,%0.2,%Δ163 | 1.7,18.4,%0.2,NA | 1.3,14.4,%0.3,%Δ28 | 0.8,8.9,%0.2,%Δ107 | 1.1,15.7,%0.2,NA | 0.9,12.7,%0.2,%Δ9 | 0.6,8.8,%0.2,%Δ77 | 0.3,6.4,%0.1,NA | 0.2,4.8,%0.1,%Δ-50 | 0.2,3.9,%0.1,%Δ-27 |
| Total Costs | 169.3,1797,NA,NA | 164.7,1765.3,NA,%Δ2 | 115.4,1231.4,NA,%Δ46 | 252.3,2404,NA,NA | 250.2,2409,NA,%Δ0 | 173.2,1659.8,NA,%Δ45 | 376.2,3585.2,NA,NA | 348.1,3352.1,NA,%Δ7 | 250.1,2397.2,NA,%Δ50 | 459.5,4378.9,NA,NA | 397.3,3825.9,%,%Δ14 | 284,2722.5,%,%Δ61 | 888.6,8468.4,NA,NA | 459.3,4422.8,NA,%Δ91 | 330.5,3168,NA,%Δ167 | 795.2,8053.1,NA,NA | 493.6,5041.5,NA,%Δ60 | 370.3,3770.2,NA,%Δ114 | 716.9,7867.8,NA,NA | 466.4,5157.9,NA,%Δ53 | 354.4,3909.2,NA,%Δ101 | 540.7,7992.1,NA,NA | 362.8,5395.3,NA,%Δ55 | 287.2,4261.7,NA,%Δ104 | 376.9,8035.6,NA,NA | 257.1,5507.1,NA,%Δ49 | 204.2,4363.9,NA,%Δ89 |

1. Year in relation to RA Diagnosis Incidence Year
2. RA Cases
3. Controls= Age- Sex- Non- Disease Matched Controls without RA
4. Random Controls = Age- Sex- Matched Controls without RA
5. Cost Categories Described in Supplement 6
6. Cost per patient (Cost in category / number of patients alive)
7. Difference in per patient cost between RA Cases and Matched Controls [(RA Group – Matched Controls or Random Matched Controls) / Matched Controls or Random Matched Controls)] * 100
