# Supplementary material for: Health care costs of rheumatoid arthritis: A longitudinal population study
Source: PLoS One. 2021 May 6;16(5):e0251334. doi: 10.1371/journal.pone.0251334 (PMC8101709; doi:10.1371/journal.pone.0251334)
Supplement: S1 File — (DOCX) [file pone.0251334.s005.docx]

**Supplement 1: Matching Variables and Description of Hopkins MEDC**

Hard Match

Year of Birth

Sex

MEDC 27 Groups (distance matrix match)

| **Admin** | **Allergy** | **Cardiovascular** | **Dental** |
| --- | --- | --- | --- |
| \| **ADM02** Surgical aftercare  **ADM03** Transplant status  **ADM05** Administrative concerns and non-specific laboratory abnormalities  **ADM06** Preventive Care \| \| --- \| | \| **ALL01** Allergic reactions  **ALL03** Allergic rhinitis  **ALL04** Asthma, w/o status asthmaticus  **ALL05** Asthma, with status asthmaticus  **ALL06** Disorders of the immune system \| \| --- \| | \| **CAR01** Cardiovascular signs and symptoms  **CAR03** Ischemic heart disease (excluding acute myocardial infarction)  **CAR04** Congenital heart disease  **CAR05** Congestive heart failure  **CAR06** Cardiac valve disorders  **CAR07** Cardiomyopathy  **CAR08** Heart murmur  **CAR09** Cardiac arrhythmia  **CAR10** Generalized atherosclerosis  **CAR11** Disorders of lipoid metabolism  **CAR12** Acute myocardial infarction  **CAR13** Cardiac arrest, shock  **CAR14** Hypertension, w/o major complications  **CAR15** Hypertension, with major complications  **CAR16** Cardiovascular disorders, other \| \| --- \| | \| **DEN01** Disorders of mouth  **DEN02** Disorders of teeth  **DEN03** Gingivitis  **DEN04** Stomatitis \| \| --- \| |
| **Ear, Nose, Throat** | **Endocrine** | **Eye** | **Female Reproductive** |
| \| **EAR01** Otitis media  **EAR02** Tinnitus  **EAR03** Temporomandibular joint disease  **EAR04** Foreign body in ears, nose, or throat  **EAR05** Deviated nasal septum  **EAR06** Otitis externa  **EAR07** Wax in ear  **EAR08** Deafness, hearing loss  **EAR09** Chronic pharyngitis and tonsillitis  **EAR10** Epistaxis  **EAR11** Acute upper respiratory tract infection  **EAR12** ENT disorders, other \| \| --- \| | \| **END02** Osteoporosis  **END03** Short stature  **END04** Hypothroidism  **END05** Other endocrine disorders  **END06** Type 2 diabetes, w/o complication  **END07** Type 2 diabetes, w/ complication  **END08** Type 1 diabetes, w/o complication  **END09** Type 1 diabetes, w/ complication \| \| --- \| | \| **EYE01** Ophthalmic signs and symptoms  **EYE02** Blindness  **EYE03** Retinal disorders (excluding diabetic retinopathy)  **EYE04** Disorders of the eyelid and lacrimal duct  **EYE05** Refractive errors  **EYE06** Cataract, aphakia  **EYE07** Conjunctivitis, keratitis  **EYE08** Glaucoma  **EYE09** Infections of eyelid  **EYE10** Foreign body in eye  **EYE11** Strabismus, amblyopia  **EYE12** Traumatic injuries of eye  **EYE13** Diabetic retinopathy  **EYE14** Eye, other disorders  **EYE15** Age-related macular degeneration \| \| --- \| | \| **FRE01** Pregnancy and delivery uncomplicated  **FRE02** Female genital symptoms  **FRE03** Endometriosis  **FRE04** Pregnancy and delivery with complications  **FRE05** Female infertility  **FRE06** Abnormal pap smear  **FRE07** Ovarian cyst  **FRE08** Vaginitis, vulvitis, cervicitis  **FRE09** Menstrual disorders  **FRE10** Contraception  **FRE11** Menopausal symptoms  **FRE12** Utero-vaginal prolapse  **FRE13** Female gynecologic conditions other.  **FRE14** Pregnancy with termination \| \| --- \| |
| **Gastrointestinal/Hepatic** | **General Signs and Symptoms** | **General Surgery** | **Genetic** |
| **GAS01** Gastrointestinal signs and symptoms  **GAS02** Inflammatory bowel disease  **GAS03** Constipation  **GAS04** Acute hepatitis  **GAS05** Chronic liver disease  **GAS06** Peptic ulcer disease  **GAS07** Gastroenteritis  **GAS08** Gastroesophageal reflux  **GAS09** Irritable bowel syndrome  **GAS10** Diverticular disease of colon  **GAS11** Acute pancreatitis  **GAS12** Chronic pancreatitis  **GAS13** Lactose intolerance  **GAS14** Gastrointestinal/Hepatic disorders, other | **GSI01** Nonspecific signs and symptoms  **GSI02** Chest pain  **GSI03** Fever  **GSI04** Syncope  **GSI05** Nausea, vomiting  **GSI06** Debility and undue fatigue  **GSI07** Lymphadenopathy  **GSI08** Edema | **GSU01** Anorectal conditions  **GSU02** Appendicitis  **GSU03** Benign and unspecified neoplasm  **GSU04** Cholelithiasis, cholecystitis  **GSU05** External abdominal hernias, hydroceles  **GSU06** Chronic cystic disease of the breast  **GSU07** Other breast disorders  **GSU08** Varicose veins of lower extremities  **GSU09** Nonfungal infections of skin and subcutaneous tissue  **GSU10** Abdominal pain  **GSU11** Peripheral vascular disease  **GSU12** Burns – 1st degree  **GSU13** Aoritc aneurysm  **GSU14** Gastrointestinal obstruction /perforation | **GTC01** Chromosomal anomalies **GTC02** Inherited metabolic disorders |
| **Genito-urinary** | **Hematologic** | **Infections** | **Malignancies** |
| **GUR01** Vesicoureteral reflux  **GUR02** Undescended testes  **GUR03** Hypospadias, other penile anomalies  **GUR04** Prostatic hypertrophy  **GUR05** Stricture of urethra  **GUR06** Urinary symptoms  **GUR07** Other male genital disease  **GUR08** Urinary tract infections  **GUR09** Renal calculi  **GUR10** Prostatitis  **GUR11** Incontinence  **GUR12** Genito-urinary disorders, other | **HEM01** Other hemolytic anemia  **HEM02** Iron deficiency, other deficiency anemias  **HEM03** Thrombophlebitis  **HEM04** Neonatal jaundice  **HEM05** Aplastic anemia  **HEM06** Deep vein thrombosis  **HEM07** Hemophilia, coagulation disorder  **HEM08** Hematologic disorders, other  **HEM09** Sickle cell disease | **INF01** Tuberculosis infection  **INF02** Fungal infections  **INF03** Infectious mononucleosis  **INF04** HIV, AIDS  **INF05** Sexually transmitted diseases  **INF06** Viral syndromes  **INF07** Lyme disease  **INF08** Septicemia  **INF09** Infections, other | **MAL01** Malignant neoplasms of the skin  **MAL02** Low impact malignant neoplasms  **MAL03** High impact malignant neoplasms  **MAL04** Malignant neoplasms, breast  **MAL05** Malignant neoplasms, cervix, uterus  **MAL06** Malignant neoplasms, ovary  **MAL07** Malignant neoplasms, esophagus  **MAL08** Malignant neoplasms, kidney  **MAL09** Malignant neoplasms, liver and biliary tract  **MAL10** Malignant neoplasms, lung  **MAL11** Malignant neoplasms, lymphomas  **MAL12** Malignant neoplasms, colorectal  **MAL13** Malignant neoplasms, pancreas  **MAL14** Malignant neoplasms, prostate  **MAL15** Malignant neoplasms, stomach  **MAL16** Acute leukemia  **MAL18** Malignant neoplasms, bladder |
| **Muskuloskeletal** | **Neonatal** | **Neurologic** | **Nutrition** |
| **MUS01** Musculoskeletal signs and symptoms **MUS02** Acute sprains and strains  **MUS03** Degenerative joint disease  **MUS04** Fractures (excluding digits)  **MUS05** Torticollis  **MUS06** Kyphoscoliosis  **MUS07** Congenital hip dislocation  **MUS08** Fractures and dislocations/digits only **MUS09** Joint disorders, trauma related  **MUS10** Fracture of neck of femur (hip)  **MUS11** Congenital anomalies of limbs, hands, and feet  **MUS12** Acquired foot deformities  **MUS13** Cervical pain syndromes  **MUS14** Low back pain  **MUS15** Bursitis, synovitis, tenosynovitis  **MUS16** Amputation status  **MUS17** Musculoskeletal disorders, other | **NEW01** Newborn status, uncomplicated  **NEW02** Newborn status, complicated  **NEW03** Low birth weight  **NEW04** Prematurity  **NEW05** Disorders of newborn period | **NUR01** Neurologic signs and symptoms  **NUR02** Headaches  **NUR03** Peripheral neuropathy, neuritis  **NUR04** Vertiginous syndromes  **NUR05** Cerebrovascular disease  **NUR06** Parkinson's disease  **NUR07** Seizure disorder  **NUR08** Multiple sclerosis  **NUR09** Muscular dystrophy  **NUR10** Sleep problems  **NUR11** Dementia and delirium  **NUR12** Quadriplegia and paraplegia  **NUR15** Head injury  **NUR16** Spinal cord injury/disorders  **NUR17** Paralytic syndromes, other  **NUR18** Cerebral palsy  **NUR19** Developmental disorder  **NUR20** Central nervous system infections **NUR21** Neurologic disorders, other  **NUR22** Migraines | **NUT01** Failure to thrive  **NUT02** Nutritional deficiencies  **NUT03** Obesity  **NUT04** Nutritional disorders, other |
| **Psychosocial/Mental Health** | **Reconstructive** | **Renal** | **Respiratory** |
| **PSY01** Anxiety, neuroses  **PSY02** Substance use  **PSY03** Tobacco use  **PSY04** Behavior problems  **PSY05** Attention deficit disorder  **PSY06** Family and social problems  **PSY07** Schizophrenia and affective psychosis  **PSY08** Personality disorders  **PSY09** Depression  **PSY10** Psychologic signs and symptoms  **PSY11** Psychosocial disorders, other  **PSY12** Bipolar disorder | **REC01** Cleft lip and palate  **REC02** Lacerations  **REC03** Chronic ulcer of the skin  **REC04** Burns--2nd and 3rd degree | **REN01** Chronic renal failure  **REN02** Fluid/electrolyte disturbances  **REN03** Acute renal failure  **REN04** Nephritis, nephrosis  **REN05** Renal disorders, other | **RES01** Respiratory signs and symptoms  **RES02** Acute lower respiratory tract infection **RES03** Cystic fibrosis  **RES04** Emphysema, chronic bronchitis, COPD  **RES05** Cough  **RES06** Sleep apnea  **RES07** Sinusitis  **RES08** Pulmonary embolism  **RES09** Tracheostomy  **RES10** Respiratory failure  **RES11** Respiratory disorders, other |
| **Rheumatologic** | **Skin** | **Toxic Effects and Adverse Events** |  |
| **RHU01** Autoimmune and connective tissue diseases  **RHU02** Gout  **RHU03** Arthropathy  **RHU04** Raynaud's syndrome  **RHU05** Rheumatoid arthritis | **SKN01** Contusions and abrasions  **SKN02** Dermatitis and eczema  **SKN03** Keloid  **SKN04** Acne  **SKN05** Disorders of sebaceous glands  **SKN06** Sebaceous cyst  **SKN07** Viral warts and molluscum contagiosum  **SKN08** Other inflammatory conditions of skin  **SKN09** Exanthems  **SKN10** Skin keratoses  **SKN11** Dermatophytoses  **SKN12** Psoriasis  **SKN13** Disease of hair and hair follicles  **SKN14** Pigmented nevus  **SKN15** Scabies and pediculosis  **SKN16** Diseases of nail  **SKN17** Other skin disorders  **SKN18** Benign neoplasm of skin and subcutaneous tissues  **SKN19** Impetigo  **SKN20** Dermatologic signs and symptoms | **TOX01** Toxic effects of nonmedicinal agents  **TOX02** Adverse effects of medicinal agents **TOX03** Adverse events from medical/surgical procedures  **TOX04** Complications of mechanical devices |  |

**Description of Expanded Diagnostic Clusters (EDC) from Hopkins ACG Manual Version 10**

**Expanded Diagnosis Clusters (EDCs) - A tool for identifying specific morbidity and removing practitioner coding variability.**

Expanded Diagnosis Clusters, or EDCs, complement the unique person-oriented approach that underpins the ACG System. EDCs are a tool that allow for the easy identification of people with specific diseases or symptoms. The EDC methodology assigns diagnostic codes found in claims or encounter data to one of 264 EDCs, which are further organized into 27 categories called Major Expanded Diagnosis Clusters (MEDCs). As broad groupings of diagnosis codes, EDCs help to account for differences in coding behavior between practitioners. MEDCs may be further aggregated into five MEDC types (Administrative, Medical, Surgical, Obstetric/Gynecologic, Psychosocial) providing a concise way of summarizing all diagnosis codes.

As a stand-alone tool, EDCs can be used to select patients with a specific condition or combination of conditions, as well as to compare the distribution of conditions in one population with another. In addition, EDCs enable tracking of disease prevalence over time. When combined with ACGs, the result is a powerful combination tool for demonstrating variability of cost within disease categories. This is useful for many profiling applications and can help to target individuals for case-management purposes.
