# Supplementary material for: Health care costs of rheumatoid arthritis: A longitudinal population study
Source: PLoS One. 2021 May 6;16(5):e0251334. doi: 10.1371/journal.pone.0251334 (PMC8101709; doi:10.1371/journal.pone.0251334)
Supplement: S2 File — (DOCX) [file pone.0251334.s006.docx]

**Supplement 3: Costing Categories and Timeline** **Data (and price) Availability for Cost Calculation**

| **Cost Outcomes** | 2001 | **2002** | **2003** | **2004** | **2005** | **2006** | **2007** | **2008** | **2009** | **2010** | **2011-2018** |
| --- | --- | --- | --- | --- | --- | --- | --- | --- | --- | --- | --- |
| Inpatient hospitalization | √ | √ | √ | √ | √ | √ | √ | √ | √ | √ | √ |
| Hospital outpatient clinic |  |  |  |  |  | √ | √ | √ | √ | √ | √ |
| Same Day Surgery |  |  | √ | √ | √ | √ | √ | √ | √ | √ | √ |
| Emergency Departments |  |  | √ | √ | √ | √ | √ | √ | √ | √ | √ |
| Dialysis Clinics |  |  |  |  |  | √ | √ | √ | √ | √ | √ |
| Oncology Clinics |  |  |  |  |  | √ | √ | √ | √ | √ | √ |
| Ontario Drug Benefits | √ | √ | √ | √ | √ | √ | √ | √ | √ | √ | √ |
| Rehabilitation |  | √ | √ | √ | √ | √ | √ | √ | √ | √ | √ |
| Complex Continuing Care |  | √ | √ | √ | √ | √ | √ | √ | √ | √ | √ |
| Home Care Services |  | ** | √ | √ | √ | √ | √ | √ | √ | √ | √ |
| Physician Billings | √ | √ | √ | √ | √ | √ | √ | √ | √ | √ | √ |
| Lab Billings (L-codes) |  | √ | √ | √ | √ | √ | √ | √ | √ | √ | √ |
| Non-Physician Billings (e.g. physiotherapists, optometrists, chiropractors) |  | √ | √ | √ | √ | √ | √ | √ | √ | √ | √ |
| Capitation (Family Health Teams) |  |  |  |  | @ | @ | √ | √ | √ | √ | √ |
| Long Term Care |  | * | * | * | * | * | * | * | √ | √ | √ |
| OMHRS (Mental Health Care Beds) |  |  |  |  |  | √ | √ | √ | √ | √ | √ |
| New Drug Funding Program |  | √ | √ | √ | √ | √ | √ | √ | √ | √ | √ |
| Assisted Device Program |  |  | √ | √ | √ | √ | √ | √ | √ | √ |  |

**Use 2003 price of each home-care services for 2002.

@Available for FHO only from May 1, 2005 to September 30, 2006 and then for both FHO and FHN since October 1, 2006

*Use OHIP and ODB to estimate the length of stay in LTC for the years preceding 2009/10. Then CCRS-LTC sector data is used for 2009/10 and onwards
